# Supplementary figures and images for: Metabolically healthy obesity, transition to unhealthy metabolic status, and vascular disease in Chinese adults: A cohort study
Source: PLoS Med. 2020 Oct 30;17(10):e1003351. doi: 10.1371/journal.pmed.1003351 (PMC7598496; doi:10.1371/journal.pmed.1003351)

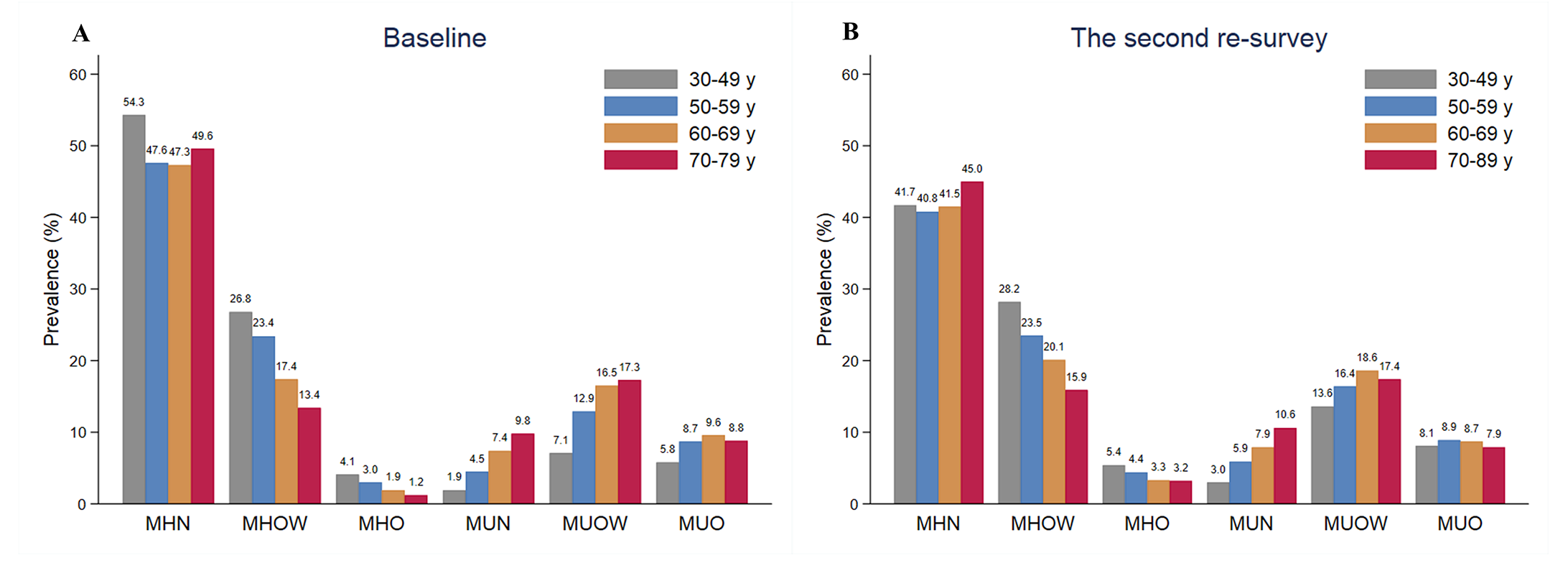

Supplement: S1 Fig — (A) Baseline; (B) the second resurvey. Prevalence by age was adjusted for sex and region. MHN, metabolically healthy normal weight; MHO, metabolically healthy obesity; MHOW, metabolically healthy overweight; MUN, metabolically unhealthy normal weight; MUO, metabolically unhealthy obesity; MUOW, metabolically unhealthy overweight. (TIF) [file pmed.1003351.s001.tif]

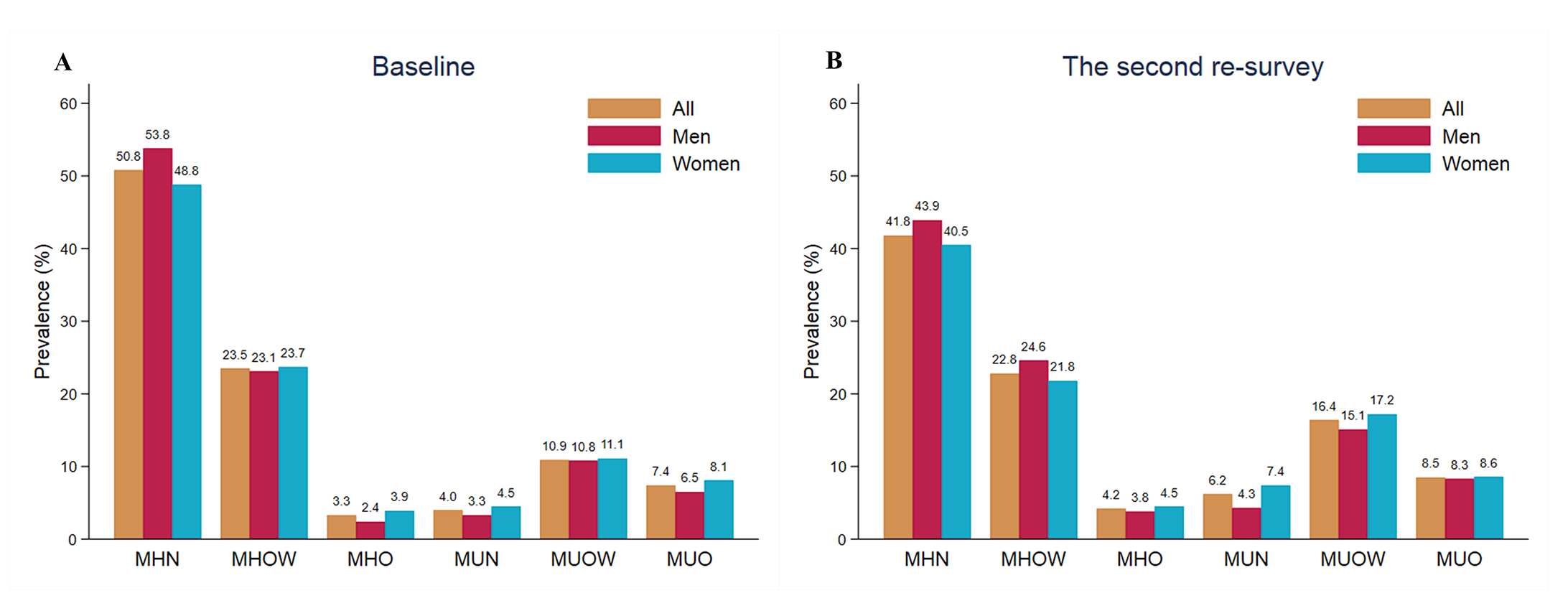

Supplement: S2 Fig — (A) Baseline; (B) The second resurvey. Prevalence by sex was adjusted for age and region. MHN, metabolically healthy normal weight; MHO, metabolically healthy obesity; MHOW, metabolically healthy overweight; MUN, metabolically unhealthy normal weight; MUO, metabolically unhealthy obesity; MUOW, metabolically unhealthy overweight. (TIF) [file pmed.1003351.s002.tif]

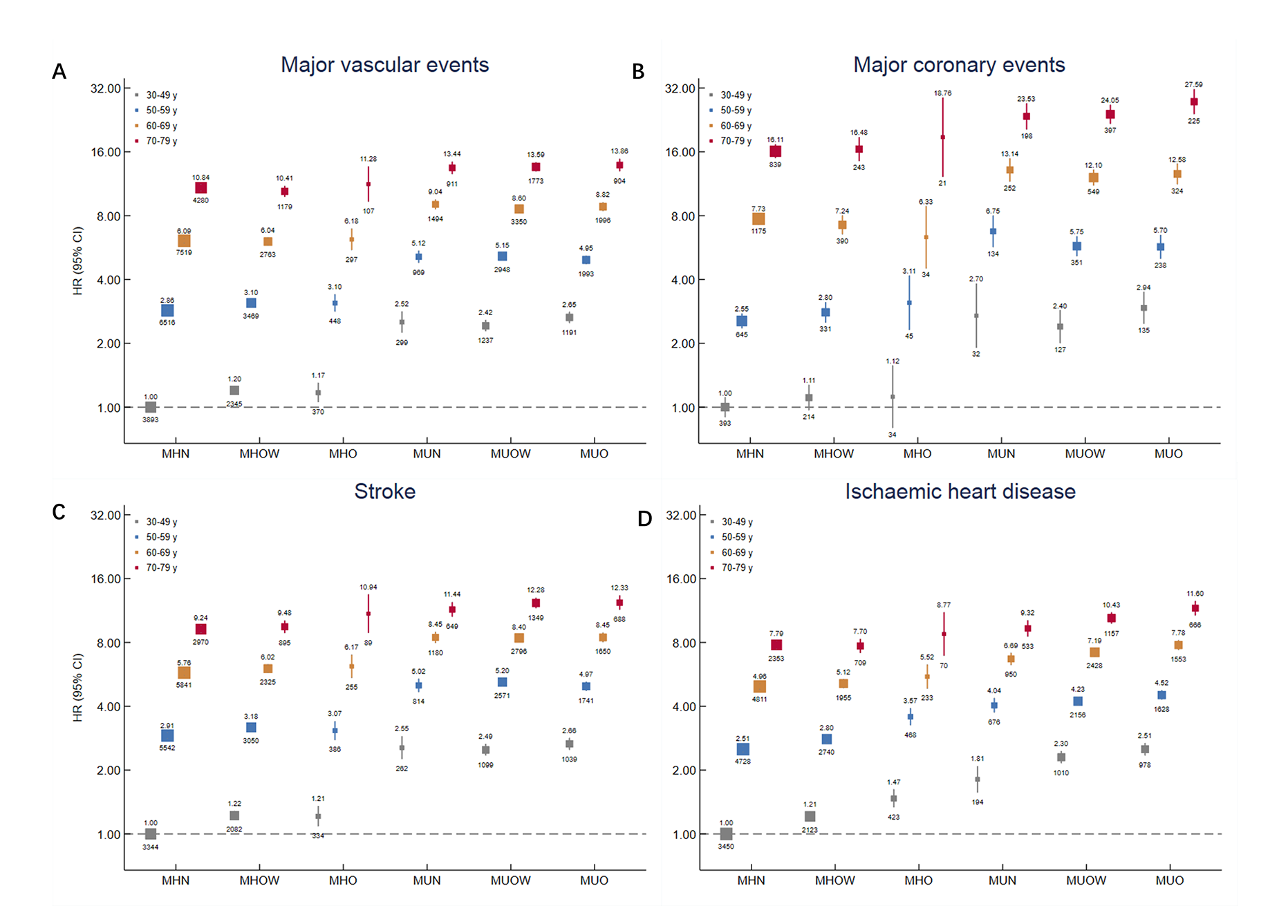

Supplement: S3 Fig — Values shown are the HR (95% CI) for (A) major vascular events, (B) major coronary events, (C) stroke, and (D) ischemic heart disease, by BMI-metabolic health status and age. HRs are adjusted for study region, sex, education, household income, marital status, smoking, alcohol use, red meat intake, fresh fruits intake, fresh vegetables intake, physical activity, and family history of heart attack or stroke. The vertical lines indicate 95% CIs. The values above the squares indicate HRs and the values under the squares indicate number of cases in each category. The size of the squares is proportional to the inverse variance of each effect size. BMI, body mass index; CI, confidence interval; HR, hazard ratio; MHN, metabolically healthy normal weight; MHO, metabolically healthy obesity; MHOW, metabolically healthy overweight; MUN, metabolically unhealthy normal weight; MUO, metabolically unhealthy obesity; MUOW, metabolically unhealthy overweight. (TIF) [file pmed.1003351.s003.tif]

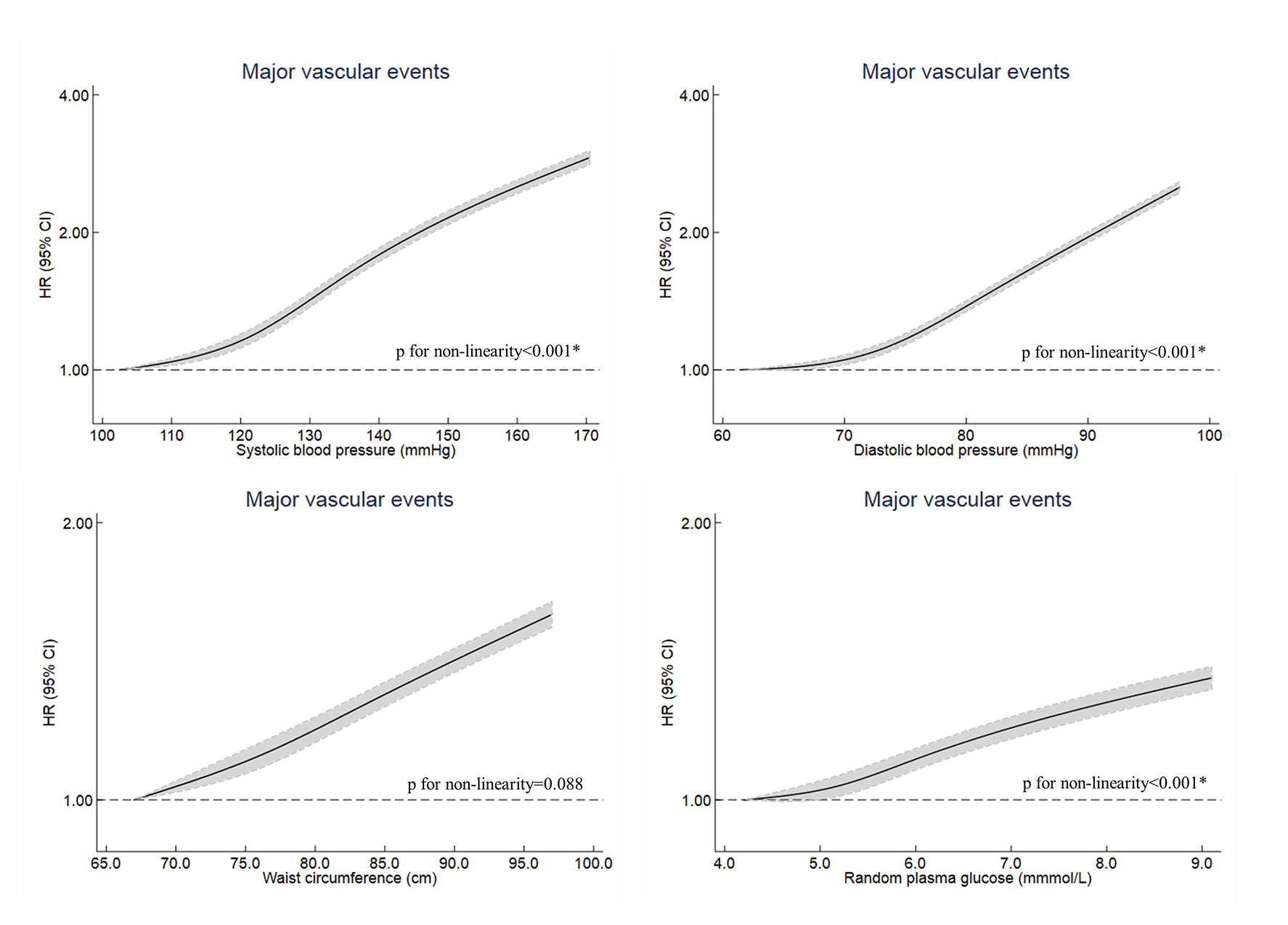

Supplement: S4 Fig — Results are adjusted for study region, sex, education, household income, marital status, smoking status, alcohol use, red meat intake, fresh fruits intake, fresh vegetables intake, physical activity, and family history of heart attack or stroke. *p < 0.05, significant nonlinear relationship. (TIF) [file pmed.1003351.s004.tif]

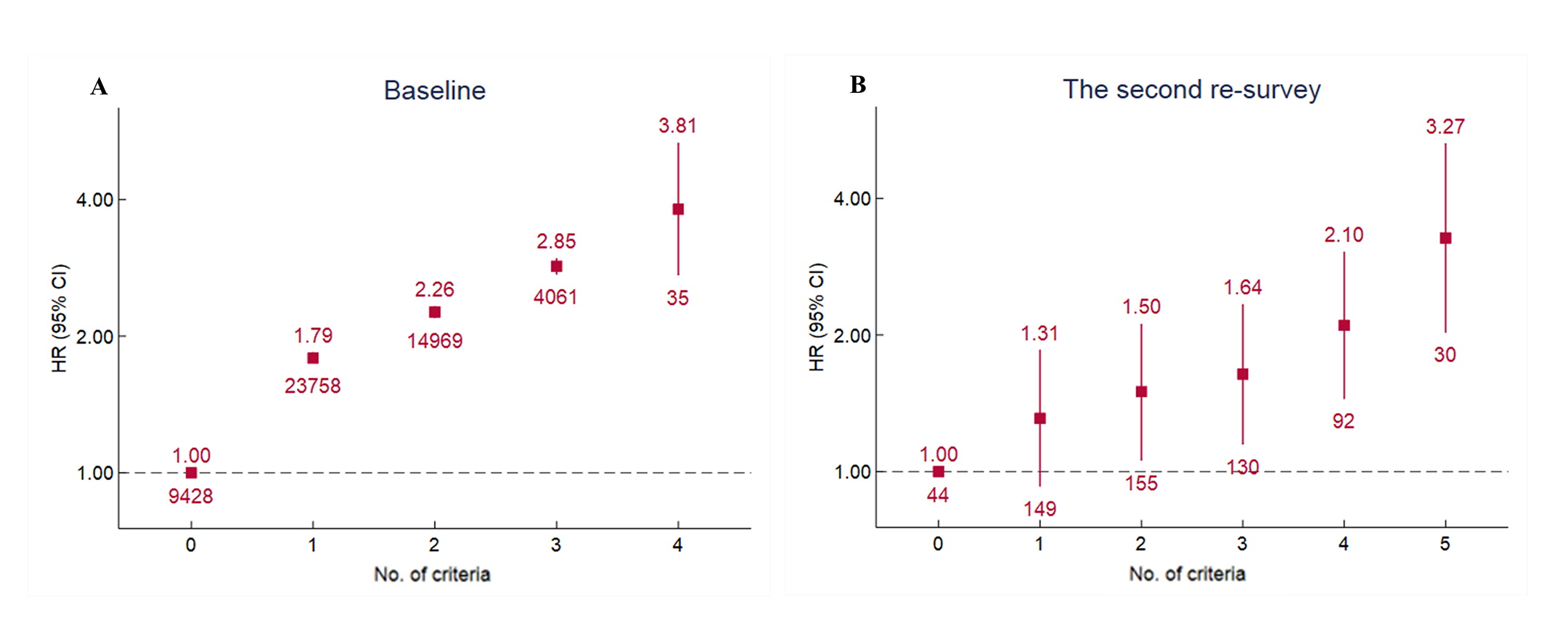

Supplement: S5 Fig — Values shown are the HR (95% CI) for major vascular events by the number of criteria of metabolic disorders participants met (A) at baseline, and (B) in the second resurvey. HRs are adjusted for study region, sex, education, household income, marital status, smoking, alcohol use, red meat intake, fresh fruits intake, fresh vegetables intake, physical activity, and family history of heart attack or stroke. The vertical lines indicate 95% CIs. The values above the squares indicate HRs and the values under the squares indicate number of cases in each category. p for trend <0.05 at baseline and the second resurvey. CI, confidence interval; HR, hazard ratio. (TIF) [file pmed.1003351.s005.tif]
