# Supplementary material for: Metabolically healthy obesity, transition to unhealthy metabolic status, and vascular disease in Chinese adults: A cohort study
Source: PLoS Med. 2020 Oct 30;17(10):e1003351. doi: 10.1371/journal.pmed.1003351 (PMC7598496; doi:10.1371/journal.pmed.1003351)
Supplement: S1 Table — (DOCX) [file pmed.1003351.s006.docx]

**S1 Table. Characteristics of the study population in the second re-survey**

| **Characteristics** | **All** | **MHN** | **MHOW** | **MHO** | **MUN** | **MUOW** | **MUO** |
| --- | --- | --- | --- | --- | --- | --- | --- |
| No. of participants | 16,225 | 6,780 | 3,707 | 687 | 1,013 | 2,658 | 1,380 |
| **Demographic factors** |  |  |  |  |  |  |  |
| Age (y) | 37.5 | 39.2 | 40.0 | 33.9 | 26.9 | 34.9 | 36.8 |
| Male (%) | 38.9 | 34.0 | 44.3 | 44.5 | 35.5 | 41.8 | 42.1 |
| Urban (%) | 37.5 | 39.2 | 40.0 | 33.9 | 26.9 | 34.9 | 36.8 |
| **Socioeconomic factors (%)** |  |  |  |  |  |  |  |
| Middle school and above | 47.9 | 48.0 | 48.6 | 44.2 | 50.2 | 47.4 | 47.3 |
| Household income≥20,000 yuan/year | 78.4 | 78.0 | 79.7 | 77.7 | 75.7 | 79.1 | 78.0 |
| Married | 89.0 | 88.4 | 89.4 | 89.1 | 89.5 | 89.5 | 89.3 |
| **Lifestyle factors** |  |  |  |  |  |  |  |
| Current smoker (%) | 23.3 | 24.8 | 21.2 | 20.0 | 26.7 | 21.9 | 23.8 |
| Current smoker-male (%) | 59.2 | 62.9 | 54.2 | 51.9 | 65.8 | 55.6 | 60.8 |
| Current smoker-female (%) | 1.8 | 2.6 | 1.9 | 1.2 | 2.8 | 1.6 | 1.5 |
| Weekly drinker (%) | 12.5 | 12.8 | 12.0 | 12.2 | 11.1 | 12.1 | 13.6 |
| Weekly drinker, male (%) | 30.0 | 30.6 | 29.2 | 26.7 | 28.0 | 29.7 | 33.0 |
| Weekly drinker, female (%) | 1.9 | 2.2 | 1.7 | 3.3 | 1.1 | 1.5 | 2.0 |
| Physical activity (MET-h/d) | 19.6 (13.9) | 20.3 (14.6) | 20.1 (13.7) | 19.8 (13.4) | 18.5 (13.0) | 18.3 (13.1) | 17.8 (12.7) |
| Meat intake (day/week) | 4.2 (2.7) | 4.2 (2.8) | 4.3 (2.7) | 4.3 (2.7) | 4.1 (2.9) | 4.2 (2.8) | 4.2 (2.7) |
| Vegetable intake (day/week) | 6.9 (0.6) | 6.9 (0.7) | 6.9 (0.5) | 6.9 (0.8) | 6.9 (0.7) | 6.9 (0.7) | 6.9 (0.7) |
| Fruit intake (day/week) | 3.6 (2.7) | 3.5 (2.7) | 3.7 (2.7) | 3.7 (2.6) | 3.5 (2.8) | 3.6 (2.7) | 3.6 (2.7) |
| **Physical measurements** |  |  |  |  |  |  |  |
| BMI (kg/m^2^) | 24.4 (3.2) | 21.7 (1.4) | 25.5 (1.1) | 29.7 (1.9) | 22.5 (1.2) | 26.0 (1.1) | 30.2 (2.0) |
| WC (cm) | 84.8 (9.3) | 77.7 (6.0) | 86.6 (5.7) | 96.4 (7.0) | 83.2 (6.1) | 90.7 (5.2) | 98.6 (7.2) |
| Waist-hip ratio | 0.9 (0.1) | 0.9 (0.1) | 0.9 (0.1) | 0.9 (0.1) | 0.9 (0.1) | 0.9 (0.1) | 1.0 (0.1) |
| SBP (mmHg) | 135.4 (20.2) | 130.6 (19.5) | 133.2 (19.2) | 137.0 (20.3) | 142.8 (18.7) | 142.7 (18.8) | 144.8 (18.9) |
| DBP (mmHg) | 78.4 (10.9) | 75.3 (10.2) | 77.9 (10.2) | 81.0 (10.5) | 80.8 (10.8) | 82.5 (10.6) | 84.5 (11.1) |
| **Self-reported conditions (%)** |  |  |  |  |  |  |  |
| Elevated WC | 40.3 | 4.8 | 43.7 | 92.5 | 33.9 | 84.7 | 98.7 |
| Elevated BP | 62.2 | 48.9 | 53.3 | 60.7 | 89.9 | 86.4 | 88.2 |
| Elevated plasma glucose | 11.7 | 4.8 | 4.0 | 2.0 | 35.0 | 25.6 | 27.3 |
| Elevated TG | 36.9 | 17.8 | 19.5 | 12.1 | 86.1 | 78.0 | 75.3 |
| Reduced HDL-C | 36.9 | 21.4 | 24.5 | 13.3 | 76.5 | 68.3 | 68.1 |
| **Family medical history (%)** |  |  |  |  |  |  |  |
| Stroke | 19.6 | 19.2 | 19.7 | 19.5 | 19.2 | 19.5 | 21.7 |
| Heart attack | 5.0 | 4.8 | 5.1 | 6.2 | 5.3 | 4.9 | 5.4 |

The second re-survey characteristics of the study population were described adjusted for age, sex and region except for number of participants, age, sex and urban region.

BMI, body mass index; BP, blood pressure; DBP, diastolic blood pressure; HDL-C, high-density lipoprotein cholesterol; MET-h/d, metabolic equivalents of task per hours per day; MHN, metabolically healthy normal weight; MHO, metabolically healthy obesity; MHOW, metabolically healthy overweight; MUN, metabolically unhealthy normal weight; MUO, metabolically unhealthy obesity; MUOW, metabolically unhealthy overweight; SBP, systolic blood pressure; TG, triglycerides; WC, waist circumference.
