# Supplementary material for: Metabolically healthy obesity, transition to unhealthy metabolic status, and vascular disease in Chinese adults: A cohort study
Source: PLoS Med. 2020 Oct 30;17(10):e1003351. doi: 10.1371/journal.pmed.1003351 (PMC7598496; doi:10.1371/journal.pmed.1003351)
Supplement: S2 Table — BMI, body mass index. (DOCX) [file pmed.1003351.s007.docx]

**S2 Table. Adjusted hazard ratios for vascular diseases by BMI-metabolic health status in the second re-survey**

|  | **MHN** | **MHOW** | **MHO** | **MUN** | **MUOW** | **MUO** |
| --- | --- | --- | --- | --- | --- | --- |
| **Major vascular events** | | |  |  |  |  |
| Cases | 222 | 110 | 16 | 49 | 138 | 65 |
| Person-years | 19,564 | 10,870 | 2,056 | 2,811 | 7,576 | 3,970 |
| HR, model 1 | 1.00 (0.87-1.14) | 1.06 (0.87-1.27) | 0.84 (0.51-1.37) | 1.31 (0.99-1.74) | 1.64 (1.38-1.93) | 1.56 (1.22-1.99) |
| HR, model 2 | 1.00 (0.87-1.14) | 1.09 (0.90-1.31) | 0.84 (0.52-1.38) | 1.25 (0.94-1.66) | 1.61 (1.36-1.90) | 1.50 (1.18-1.92) |
| **Major coronary events** | |  |  |  |  |  |
| Cases | 19 | 9 | 2 | 9 | 14 | 5 |
| Person-years | 19,785 | 10,997 | 2,077 | 2,864 | 7,740 | 4,061 |
| HR, model 1 | 1.00 (0.63-1.58) | 0.98 (0.51-1.90) | 1.24 (0.31-4.97) | 3.16 (1.63-6.14) | 1.99 (1.18-3.37) | 1.37 (0.57-3.31) |
| HR, model 2 | 1.00 (0.63-1.59) | 1.04 (0.54-2.02) | 1.32 (0.33-5.30) | 3.01 (1.54-5.87) | 2.04 (1.21-3.44) | 1.39 (0.57-3.35) |
| **Ischaemic heart disease** | |  |  |  |  |  |
| Cases | 157 | 93 | 23 | 39 | 79 | 55 |
| Person-years | 19,589 | 10,883 | 2,053 | 2,825 | 7,653 | 3,993 |
| HR, model 1 | 1.00 (0.85-1.17) | 1.18 (0.96-1.45) | 1.58 (1.05-2.38) | 1.46 (1.06-2.00) | 1.25 (1.00-1.56) | 1.75 (1.34-2.28) |
| HR, model 2 | 1.00 (0.85-1.17) | 1.20 (0.98-1.48) | 1.61 (1.07-2.43) | 1.41 (1.03-1.94) | 1.26 (1.01-1.57) | 1.76 (1.35-2.30) |
| **Stroke** |  |  |  |  |  |  |
| Cases | 194 | 99 | 12 | 38 | 119 | 59 |
| Person-years | 19,576 | 10,872 | 2,058 | 2,818 | 7,583 | 3,972 |
| HR, model 1 | 1.00 (0.87-1.15) | 1.08 (0.89-1.32) | 0.71 (0.40-1.25) | 1.15 (0.83-1.58) | 1.60 (1.34-1.91) | 1.61 (1.25-2.07) |
| HR, model 2 | 1.00 (0.87-1.15) | 1.11 (0.91-1.36) | 0.71 (0.40-1.24) | 1.09 (0.79-1.51) | 1.57 (1.31-1.88) | 1.53 (1.19-1.98) |

Multivariable models were adjusted for model 1: study region, age (5 years) and sex (men or women) and model 2: study region, age (5 years), sex (men or women), education (primary school or lower, middle school or higher), household income (<20,000 yuan/year, or ≥20,000 yuan/year), marital status (married, others), smoking status (current regular smoker, not current regular smoker), alcohol use(weekly drinker, not weekly drinker), intakes of red meat, fresh fruits and vegetables(daily, 4-6 days/week, 1-3 days/week, monthly, or never/rarely), family history of heart attack or stroke (presence or absence) and physical activity (3 groups).

BMI, body mass index; HR, hazard ratio; MHN, metabolically healthy normal weight; MHO, metabolically healthy obesity; MHOW, metabolically healthy overweight; MUN, metabolically unhealthy normal weight; MUO, metabolically unhealthy obesity; MUOW, metabolically unhealthy overweight.
