# Supplementary material for: Metabolically healthy obesity, transition to unhealthy metabolic status, and vascular disease in Chinese adults: A cohort study
Source: PLoS Med. 2020 Oct 30;17(10):e1003351. doi: 10.1371/journal.pmed.1003351 (PMC7598496; doi:10.1371/journal.pmed.1003351)
Supplement: S3 Table — BMI, body mass index. (DOCX) [file pmed.1003351.s008.docx]

**S3 Table. Sensitivity analysis of association between BMI-metabolic health and types of cardiovascular disease**

| Disease | Baseline HR (95% CI) | | | | | |
| --- | --- | --- | --- | --- | --- | --- |
|  | MHN | MHOW | MHO | MUN | MUOW | MUO |
| **Major vascular event** |  |  |  |  |  |  |
| Exclude cases occurred in the first 2 years | 1.00 (0.99-1.01) | 1.06 (1.04-1.09) | 1.10 (1.03-1.16) | 1.51 (1.46-1.56) | 1.57 (1.54-1.60) | 1.66 (1.61-1.70) |
| Never smoking | 1.00 (0.98-1.02) | 1.06 (1.03-1.09) | 1.10 (1.03-1.18) | 1.51 (1.44-1.57) | 1.57 (1.53-1.61) | 1.63 (1.58-1.68) |
| Additionally adjusted for covariates* | 1.00 (0.99-1.01) | 1.05 (1.03-1.07) | 1.08 (1.02-1.14) | 1.54 (1.49-1.59) | 1.58 (1.55-1.61) | 1.67 (1.62-1.71) |
| Additionally adjusted for covariates† | 1.00 (0.99-1.01) | 1.01 (0.99-1.03) | 1.11 (1.05-1.17) | 1.18 (1.14-1.22) | 1.21 (1.19-1.24) | 1.25 (1.21-1.28) |
| WHR instead of waist circumference | 1.00 (0.98-1.02) | 1.03 (1.00-1.06) | 1.18 (1.11-1.25) | 1.59 (1.56-1.62) | 1.66 (1.63-1.69) | 1.87 (1.82-1.91) |
| WHtR instead of waist circumference | 1.00 (0.98-1.02) | 0.91 (0.88-0.94) | 1.04 (0.98-1.12) | 1.59 (1.55-1.62) | 1.62 (1.60-1.65) | 1.81 (1.77-1.86) |
| Continuous variables instead of factor variables | 1.00 (0.99-1.01) | 1.08 (1.06-1.10) | 1.09 (1.03-1.15) | 1.64 (1.58-1.69) | 1.59 (1.56-1.62) | 1.70 (1.66-1.75) |
| Excluding waist circumference criterion | 1.00 (0.98-1.02) | 1.12 (1.08-1.16) | 1.34 (1.25-1.43) | 1.91 (1.88-1.94) | 2.08 (2.05-2.12) | 2.35 (2.29-2.41) |
| **Major coronary event** |  |  |  |  |  |  |
| Exclude cases occurred in the first 2 years | 1.00 (0.96-1.04) | 1.03 (0.97-1.09) | 1.07 (0.90-1.28) | 1.79 (1.64-1.94) | 1.75 (1.66-1.85) | 1.95 (1.82-2.09) |
| Never smoking | 1.00 (0.94-1.06) | 1.03 (0.94-1.12) | 1.19 (0.96-1.48) | 1.79 (1.61-1.99) | 1.75 (1.63-1.88) | 1.87 (1.71-2.03) |
| Additionally adjusted for covariates* | 1.00 (0.96-1.04) | 1.01 (0.96-1.07) | 1.04 (0.88-1.24) | 1.78 (1.64-1.93) | 1.71 (1.63-1.81) | 1.90 (1.78-2.03) |
| Additionally adjusted for covariates† | 1.00 (0.96-1.04) | 0.97 (0.92-1.03) | 1.08 (0.91-1.28) | 1.34 (1.23-1.45) | 1.32 (1.25-1.39) | 1.43 (1.34-1.53) |
| WHR instead of waist circumference | 1.00 (0.95-1.05) | 1.01 (0.94-1.10) | 1.28 (1.09-1.52) | 1.73 (1.65-1.82) | 1.78 (1.70-1.86) | 2.15 (2.02-2.30) |
| WHtR instead of waist circumference | 1.00 (0.96-1.04) | 0.84 (0.76-0.92) | 1.00 (0.81-1.23) | 1.74 (1.65-1.83) | 1.73 (1.66-1.81) | 2.10 (1.97-2.24) |
| Continuous variables instead of factor variables | 1.00 (0.96-1.04) | 1.08 (1.02-1.15) | 1.08 (0.91-1.28) | 1.99 (1.84-2.15) | 1.73 (1.64-1.82) | 1.88 (1.76-2.01) |
| Excluding waist circumference criterion | 1.00 (0.94-1.07) | 1.00 (0.91-1.11) | 1.25 (1.01-1.54) | 1.88 (1.81-1.96) | 2.13 (2.05-2.22) | 2.62 (2.46-2.80) |
| **Ischaemic heart disease** |  |  |  |  |  |  |
| Exclude cases occurred in the first 2 years | 1.00 (0.98-1.02) | 1.11 (1.08-1.14) | 1.38 (1.30-1.46) | 1.36 (1.30-1.43) | 1.58 (1.54-1.62) | 1.79 (1.74-1.85) |
| Never smoking | 1.00 (0.98-1.02) | 1.14 (1.11-1.18) | 1.38 (1.29-1.47) | 1.36 (1.29-1.43) | 1.55 (1.51-1.60) | 1.75 (1.69-1.81) |
| Additionally adjusted for covariates* | 1.00 (0.98-1.02) | 1.10 (1.07-1.12) | 1.34 (1.26-1.42) | 1.41 (1.35-1.46) | 1.59 (1.55-1.63) | 1.78 (1.73-1.83) |
| Additionally adjusted for covariates† | 1.00 (0.98-1.02) | 1.07 (1.05-1.10) | 1.34 (1.27-1.42) | 1.24 (1.19-1.29) | 1.41 (1.38-1.45) | 1.56 (1.52-1.61) |
| WHR instead of waist circumference | 1.00 (0.98-1.02) | 1.14 (1.11-1.18) | 1.51 (1.43-1.60) | 1.34 (1.31-1.38) | 1.52 (1.49-1.55) | 1.85 (1.80-1.91) |
| WHtR instead of waist circumference | 1.00 (0.98-1.02) | 1.08 (1.04-1.11) | 1.43 (1.34-1.52) | 1.41 (1.37-1.44) | 1.53 (1.50-1.56) | 1.86 (1.81-1.91) |
| Continuous variables instead of factor variables | 1.00 (0.98-1.02) | 1.12 (1.10-1.15) | 1.34 (1.26-1.42) | 1.48 (1.43-1.55) | 1.58 (1.54-1.62) | 1.74 (1.69-1.79) |
| Excluding waist circumference criterion | 1.00 (0.98-1.03) | 1.19 (1.15-1.23) | 1.58 (1.48-1.68) | 1.43 (1.40-1.46) | 1.67 (1.64-1.71) | 2.07 (2.01-2.13) |
| **Stroke** |  |  |  |  |  |  |
| Exclude cases occurred in the first 2 years | 1.00 (0.98-1.02) | 1.10 (1.08-1.13) | 1.13 (1.06-1.20) | 1.50 (1.44-1.56) | 1.62 (1.58-1.66) | 1.68 (1.64-1.73) |
| Never smoking | 1.00 (0.98-1.02) | 1.10 (1.07-1.13) | 1.10 (1.02-1.18) | 1.50 (1.43-1.57) | 1.61 (1.57-1.66) | 1.66 (1.60-1.71) |
| Additionally adjusted for covariates* | 1.00 (0.98-1.02) | 1.10 (1.07-1.12) | 1.11 (1.04-1.18) | 1.54 (1.48-1.59) | 1.64 (1.60-1.68) | 1.70 (1.66-1.75) |
| Additionally adjusted for covariates† | 1.00 (0.98-1.02) | 1.04 (1.02-1.07) | 1.14 (1.07-1.21) | 1.17 (1.13-1.21) | 1.25 (1.23-1.28) | 1.26 (1.23-1.30) |
| WHR instead of waist circumference | 1.00 (0.98-1.02) | 1.06 (1.03-1.09) | 1.20 (1.12-1.27) | 1.59 (1.56-1.63) | 1.73 (1.70-1.77) | 1.91 (1.86-1.96) |
| WHtR instead of waist circumference | 1.00 (0.98-1.02) | 0.94 (0.91-0.97) | 1.08 (1.00-1.16) | 1.60 (1.56-1.64) | 1.70 (1.67-1.73) | 1.86 (1.81-1.91) |
| Continuous variables instead of factor variables | 1.00 (0.98-1.02) | 1.11 (1.09-1.14) | 1.12 (1.05-1.19) | 1.63 (1.57-1.69) | 1.64 (1.60-1.68) | 1.74 (1.69-1.79) |
| Excluding waist circumference criterion | 1.00 (0.97-1.03) | 1.18 (1.14-1.22) | 1.40 (1.30-1.50) | 1.97 (1.94-2.01) | 2.21 (2.17-2.25) | 2.45 (2.38-2.51) |

* Based on model 2, additionally adjusted for the amount of cigarettes consumed per day (1-14, 15-24, ≥25) and the amount of alcohol consumed (<15, 15-29, 30-59, ≥60 g/day).

† Based on model 2, additionally adjusted for systolic blood pressure (<114, 114-123.4, 123.5-132.4, 132.5-146.4, ≥146.5 mm Hg) and random plasma glucose (<4.8, 4.8-5.2, 5.3-5.7, 5.8-6.6, ≥6.7 mmol/L)

BMI, body mass index; CI, confidence interval; HR, hazard ratio; MHN, metabolically healthy normal weight; MHO, metabolically healthy obesity; MHOW, metabolically healthy overweight; MUN, metabolically unhealthy normal weight; MUO, metabolically unhealthy obesity; MUOW, metabolically unhealthy overweight; WHR, waist-hip ratio; WHtR, waist-height ratio.
