# Supplementary material for: Metabolically healthy obesity, transition to unhealthy metabolic status, and vascular disease in Chinese adults: A cohort study
Source: PLoS Med. 2020 Oct 30;17(10):e1003351. doi: 10.1371/journal.pmed.1003351 (PMC7598496; doi:10.1371/journal.pmed.1003351)
Supplement: S4 Table — BMI, body mass index. (DOCX) [file pmed.1003351.s009.docx]

**S4 Table. Sensitivity analysis of association between changes of BMI-metabolic health and types of cardiovascular disease by using continuous variables**

|  | Stable MHN | Change from MHN to MHOO | Stable MHOO | Change from MHOO to MUOO | Stable MUOO |
| --- | --- | --- | --- | --- | --- |
| **Major vascular events** | |  |  |  |  |
| model 1 | 1.00 (0.91-1.10) | 1.15 (0.94-1.41) | 1.07 (0.93-1.24) | 1.36 (1.19-1.56) | 1.94 (1.74-2.16) |
| model 2 | 1.00 (0.91-1.10) | 1.16 (0.95-1.42) | 1.10 (0.95-1.27) | 1.39 (1.22-1.59) | 1.93 (1.73-2.15) |
| **Major coronary events** | |  |  |  |  |
| model 1 | 1.00 (0.70-1.43) | 1.48 (0.79-2.78) | 1.10 (0.65-1.86) | 1.65 (1.02-2.69) | 2.19 (1.51-3.16) |
| model 2 | 1.00 (0.70-1.43) | 1.53 (0.81-2.88) | 1.17 (0.69-1.99) | 1.71 (1.05-2.79) | 2.12 (1.46-3.08) |
| **Ischaemic heart disease** | |  |  |  |  |
| model 1 | 1.00 (0.90-1.11) | 1.07 (0.86-1.33) | 1.14 (0.98-1.32) | 1.23 (1.06-1.43) | 1.74 (1.54-1.96) |
| model 2 | 1.00 (0.90-1.11) | 1.07 (0.86-1.33) | 1.15 (0.99-1.33) | 1.23 (1.06-1.42) | 1.72 (1.52-1.94) |
| **Stroke** |  |  |  |  |  |
| model 1 | 1.00 (0.90-1.11) | 1.10 (0.89-1.36) | 1.05 (0.90-1.22) | 1.33 (1.16-1.53) | 1.91 (1.71-2.14) |
| model 2 | 1.00 (0.90-1.11) | 1.10 (0.89-1.36) | 1.07 (0.92-1.24) | 1.35 (1.18-1.56) | 1.91 (1.70-2.13) |

Multivariable models were adjusted for model 1: study region, age (5 years) and sex (men or women) and model 2: study region, age (5 years), sex (men or women), education level (primary school or lower, middle school or higher), household income (<20,000 yuan/year, or ≥20,000 yuan/year), marital status (married, others), smoking status (current regular smoker, not current regular smoker), alcohol use (weekly drinker, not weekly drinker), frequency of fruit intake, frequency of vegetable intake, frequency of meat intake (day/week), family history of heart attack or, stroke (presence or absence) and physical activity (MET-h/d).

BMI, body mass index; MET-h/d, metabolic equivalents of task per hours per day; MHN, metabolically healthy normal weight; MHOO, metabolically healthy overweight or obesity; MUOO, metabolically unhealthy overweight or obesity.
