# Supplementary material for: Metabolically healthy obesity, transition to unhealthy metabolic status, and vascular disease in Chinese adults: A cohort study
Source: PLoS Med. 2020 Oct 30;17(10):e1003351. doi: 10.1371/journal.pmed.1003351 (PMC7598496; doi:10.1371/journal.pmed.1003351)
Supplement: S1 Text — (DOCX) [file pmed.1003351.s011.docx]

**Analysis Plan**

Metabolically healthy obesity and cardiovascular diseases among Chinese: an analysis based on the China Kadoorie Biobank.

Date: December 2018

To better understand the role of metabolically healthy obesity as a risk factor for diseases with major public health relevance, we examined the association of the metabolically healthy obesity with incident cardiovascular diseases among Chinese adults by using the China Kadoorie Biobank (CKB).

**Main Exposures:** Metabolically healthy obesity. Metabolic status (defined as “healthy” or “unhealthy”) was based on five CVD risk factors: elevated waist circumference, elevated blood pressure, elevated triglyceride levels, reduced HDL cholesterol, and elevated fasting glucose.

**Main Outcomes:** Cardiovascular disease [ICD-10: I00-I99], major vascular events [ICD-10: I00-I99 (only where fatal) and I21-I23, I60, I61, I63, I64 (from any source)], major coronary events [ICD-10: I20-I25 (only where fatal) and I21-I23 (from any source)], ischaemic heart disease [ICD-10: I20-I25], and stroke [ICD-10: I60, I61, I63, and I64]. Events assessed until December 31, 2016.

**Inclusion criteria:**

Participants enrolled in CKB without cardiovascular disease or cancer.

Participants with complete information of BMI and metabolic status.

**Analysis:**

Mainly analysis methods: Cox proportional hazards model and floating absolute risk.

Time scale: age

Covariates: Age, sex, study region, education, household income, marital status, smoking status, alcohol use, intakes of red meat, fresh fruits and vegetables, physical, and family history of heart attack or stroke.

Sensitivity analysis: Excluding cases occurring in the first 2 years of follow-up; excluding ever smokers; additionally adjusting for the amount of cigarettes consumed per day and the amount of alcohol consumed.

**Not prespecified analysis:**

Sensitivity analysis: Additionally adjusting for systolic blood pressure and RPG to report whether the associations were caused by difference of blood pressure and RPG; using waist hip ratio instead of the waist circumference criterion defined metabolic health status; using waist height ratio instead of the waist circumference criterion.
