# Supplementary material for: Metabolically healthy obesity, transition to unhealthy metabolic status, and vascular disease in Chinese adults: A cohort study
Source: PLoS Med. 2020 Oct 30;17(10):e1003351. doi: 10.1371/journal.pmed.1003351 (PMC7598496; doi:10.1371/journal.pmed.1003351)
Supplement: S2 Text — (PDF) [file pmed.1003351.s012.pdf]

June 10, 2004

Kadoorie Study of Chronic Disease in China

【Questionnaire】

Clinical Trial Service Unit & Epidemiological Studies Unit,
Nuffield Department of Clinical Medicine, University of Oxford, UK
&
Chinese Centre for Disease Control and Prevention, Beijing, P. R. China

Section 1: Background information

1.1 Study ID: ☐☐☐☐☐☐☐☐☐☐ *(The date & time of interview will be recorded by computer)*

1.2 Name____________, **Sex** : Male ☐ Female ☐ , **Name of spouse**________________

1.3 Date of birth: ☐☐☐☐ Year ☐☐ Month ☐☐ Day

1.4 National ID number (if no, put #) ☐☐☐☐☐☐☐☐☐☐☐☐☐☐☐☐☐☐

1.5 Home address : _____ Province _____ City_________ District/County _____ Street/Village

Home telephone: Not available ☐ , Yes: ☐☐☐-☐☐☐☐☐☐☐☐☐☐☐

1.6 What is the highest level of school education you ever received?

- ☐ No formal school
- ☐ Primary School
- ☐ Middle School
- ☐ High School
- ☐ Technical school / college
- ☐ University

1.7 What is your current occupation?

- ☐ Agriculture & related workers
- ☐ Factory worker
- ☐ Administrator / manager
- ☐ Professional / technical
- ☐ Sales & service workers
- ☐ Retired
- ☐ House wife / husband
- ☐ Self-employed
- ☐ Unemployed
- ☐ Other or not stated

1.8 How many people living together in the household? ☐☐ persons

1.9 What is your current marital status?

- ☐ Married
- ☐ Widowed
- ☐ Separated / divorced
- ☐ Never married

1.10 What is the total income last year in your household?

- ☐ <2,500 yuan
- ☐ 2,500-4,999 yuan
- ☐ 5,000-9,999 yuan
- ☐ 10,000-19,999 yuan
- ☐ 20,000-34,999 yuan
- ☐ ≥35,000 yuan

1.11 Do you have any health care cover and following items in your household?

Yes	No	
☐	☐	Health care cover
☐	☐	Own house / apartment
☐	☐	Toilet for private use
☐	☐	Telephone or mobile phone
☐	☐	Motor vehicle (e.g. car or motorbike)
☐	☐	Holiday during last five years

Section 2: Tea drinking

2.1 During the past 12 months, how often did you drink any tea?

- ☐ Never or almost never
- ☐ Only occasionally
- ☐ Only at certain seasons
- ☐ Every month but less than weekly
- ☐ Usually at least once a week → ***Go to Q2.3*** (were2.2)

2.2(were 2.1a) **In the past, did you ever have a period of at least 1 year during which you usually drank tea at least once a week?**

- ☐ Yes, → (were 2.1b) if so, how long ago did it end? ☐☐ Years } ***Go to section 3***
- ☐ No

2.3(were 2.2) **During the past 12 months, on how many days did you drink tea in a typical week?**

- ☐ 1-2 days/week
- ☐ 3-5 days/week
- ☐ Daily or almost every day

2.4(were2.3)**At about what age did you start drinking tea in most weeks?** ……… ☐☐ Years

---

2.5(were2.4)**On days when you drink tea, how many cups do you usually drink?** (choose one only)

Green /Jasmine tea ………………………………………………………… ☐☐ cups/day

Oolong tea ………………………………………………………………… ☐☐ cups/day

Black tea ………………………………………………………………… ☐☐ cups/day

Other tea ………………………………………………………………… ☐☐ cups/day

2.6(were2.5) **How often do you change tea leaves during a day?** ………… ☐☐ times

---

2.7(were2.6) **About how much tea leaves do you usually add each time?** ☐☐ Grams

---

2.8(were2.7) **What strength of tea do you usually prefer to drink?**

- ☐ Weak
- ☐ Moderate
- ☐ Strong

2.9(were2.8) **At about what temperature do you usually drink your tea?**

- ☐ Room temperature / warm
- ☐ Hot
- ☐ Burning hot

2.10(were2.9)**Has your tea consumption changed significantly compared with that some years ago?** ☐ About the same as before, ☐ Has increased a lot, ☐ Has decreased a lot

---

Section 3: Alcohol consumption

3.1(were3.0) **Have you drunk any alcohol today?** ☐ Yes , ☐ No

3.2(were3.1)**During the past 12 months, how often did you drink any alcohol?**

☐ Never or almost never
☐ Only occasionally
☐ Only at certain seasons
☐ Every month but less than weekly
☐ Usually at least once a week → ***Go to Q3.4*** (wereQ3.2)

3.3(were3.1a)**In the past, did you ever have a period of at least 1 year, during which you usually drank some alcohol at least once a week?**

☐ Yes, →(were 3.1b) If so, how long ago did it e[illegible] ☐☐ Years } ***Go to section 4***
☐ No

3.4(were3.2)**During the past 12 months, on how many days did you drink alcohol in a typical week?**

☐ 1-2 days/week
☐ 3-5 days/week
☐ Daily or almost every day

3.5(were3.3)**At about what age did you start drinking some alcohol in most weeks?** ☐☐ Years

---

3.6(were3.4) **On days when you drink, how much alcohol do you usually drink in a day?**
(Can choose up to 3 types of alcohol for special occasions; for beer, 1 large bottle=2 small ones)

Alcohol type	On a typical day (choose one)		On a special day when you drink a lot		Last time when you drank	
Beer (large)	☐☐	Bottle	☐☐	Bottle	☐☐	Bottle
Rice Wine	☐☐	liang	☐☐	liang	☐☐	liang
Wine	☐☐	liang	☐☐	liang	☐☐	liang
Spirit (≥50% alcohol)	☐☐	liang	☐☐	liang	☐☐	liang
Spirit (<50% alcohol)	☐☐	liang	☐☐	liang	☐☐	liang

3.7(were3.5)**On a typical day when you drink alcohol, when do you usually take the drink?**

☐ Usually drink with the meal
☐ Usually drink between or after the meals
☐ No regular pattern

3.8(were3.6)**After drinking alcohol, do you usually experience hot flushes or dizziness?**

☐ Yes, soon after first mouthful
☐ Yes, after drinking small amount of alcohol
☐ Yes, but only after drinking large amount of alcohol
☐ No

3.9(were3.7)**During the past month, how often have you drunk alcohol in the morning?**

- ☐ Never
- ☐ <1 day/week
- ☐ A few days a week
- ☐ Daily or almost daily

3.10(were3.8)**During the past month, have you ever had the following experiences?**

Yes	No	
☐	☐	Unable to work or to do anything because of drinking
☐	☐	Felt depressed, irritated or couldn't control yourself after drinking
☐	☐	Could not keep away from drinking
☐	☐	Had shakes when you stopped drinking

3.11(were3.9) **Has your alcohol consumption changed significantly compared with that some years ago?**

- ☐ About the same as before
- ☐ Has increased a lot
- ☐ Has decreased a lot

Section 4: Smoking history

4.1(were4.0) **Have you smoked any tobacco today?☐ Yes , ☐ No,** →if yes, how many: __ total, __in last hour

4.2(were4.1)**How often do you smoke tobacco now?**

- ☐ Do not smoke now
- ☐ Only occasionally
- ☐ Yes, on most days
- ☐ Yes, daily or almost every day } → ***Go to Q4.7*** (were Q4.5)

4.3(were4.2)**In the past, how frequently did you smoke?**

- ☐ Did not smoke
- ☐ Smoked only occasionally
- ☐ Smoked on most days
- ☐ Smoked daily or almost every day } → ***Go to Q 4.5*** (were Q4.3)

4.4(were4.2a)**In your life time, have you smoked a total of at least 100 cigarettes or equivalent?**

- ☐ Yes
- ☐ No } ➔ ***Please go to section 5***

4.5(were4.3)**How many years ago did you last stop smoking regularly?** ☐☐ Years

4.6(were4.4)**What was your main reason for stopping?**

- ☐ Physical illness that you already had
- ☐ Money
- ☐ Health concerns (about future illness)
- ☐ Family against
- ☐ Other

4.7(were4.5)**At about what age did you first start smoking on most days?** ☐☐ Years

4.8(were4.6)**What tobacco did you use when you first started smoking on most days?**

Mainly cigarette ☐, Mainly non-cigarette ☐, Mixed types ☐

↳ (4.6a) If so, have you always smoked some cigarettes on most days, never having a month or more without them? Yes ☐, No ☐

4.9(were4.7)**How much tobacco do you usually smoke (or did you smoke before giving up)?**

Filter cigarettes (factory)	☐☐	number/day
Non-filter cigarettes (factory)	☐☐	number/day
Hand-rolled cigarettes	☐☐	liang/month
Pipe or water pipe	☐☐	liang/month
Cigars	☐☐	number/day

4.10(were4.9) **How deeply do you usually inhale the smoke?**

- ☐ Mouth only
- ☐ Throat
- ☐ Lung ➔ 4.9a Have you always inhaled the smoke into your lung when smoking? Yes ☐, No ☐

4.11(were4.10)**Has your tobacco consumption changed significantly compared with that some years ago?**

☐ About the same as before, ☐ Has increased a lot, ☐ Has decreased a lot

Section 5: Diet

5.1(were5.2)**During the past 12 months, about how often did you eat the following foods?**

	Daily	4-6 days per week	1-3 days per week	Monthly	Never/rarely
Rice	☐	☐	☐	☐	☐
Wheat	☐	☐	☐	☐	☐
Other staple food (corn, millet etc.)	☐	☐	☐	☐	☐
Meat	☐	☐	☐	☐	☐
Poultry	☐	☐	☐	☐	☐
Fish/sea food	☐	☐	☐	☐	☐
Fresh eggs	☐	☐	☐	☐	☐
Fresh vegetables	☐	☐	☐	☐	☐
Soybean products	☐	☐	☐	☐	☐
Preserved vegetables	☐	☐	☐	☐	☐
Fresh fruit	☐	☐	☐	☐	☐
Dairy products (milk, yogurt)	☐	☐	☐	☐	☐

5.2(were5.3)**During the past 12 months, have you taken the following supplements regularly?**

Yes	No	
☐	☐	Fish oil/cod liver oil
☐	☐	Vitamins
☐	☐	Calcium/iron/zinc
☐	☐	Ginshen (at least 5 or more times during a year)
☐	☐	Other herbal products

5.3(were5.3a)**Have you ever experienced any severe food shortage?** ☐Yes, ☐ No → ***Go to Q5.6(wereQ5.3c)***

5.4(were5.3a1)**What year was the worst food shortage you experienced?** _____________ **years**

5.5(were5.3b)**During the most severe food shortage you experienced:**

5.5.1– did you lose weight? ☐ Yes, ☐ No, → If yes, about how much? _____ *jin,*

5.5.2– did you develop any specific disease related to food shortage? ☐ Yes, ☐ No

5.6(were5.3c)**How many years have you had a refrigerator in your home?** [ ][ ] Years

5.7(were5.4) **During the past month, about how often did you eat hot spicy food?**

☐ Never or almost never
☐ Only occasionally } → ***Go to section 6***
☐ 1-2 days/week
☐ 3-5 days/week
☐ Daily or almost every day

5.8(were5.5)**At what age did you start to eat spicy food at least once a week?** [ ][ ] Years

5.9(were5.6)**What strength of spicy food do you usually prefer to eat?**

☐ Weak, ☐ Moderate, ☐ Strong

5.10(were5.7)**On day when you eat spicy food, what are the main sources of spice usually used?**

Yes	No	
☐	☐	Chili sauce
☐	☐	Chili oil
☐	☐	Dried chili pepper
☐	☐	Fresh chili pepper
☐	☐	Other or don't know

Section 6: Passive smoking & indoor air pollution

6.1 Have you ever lived with smoker in the same house for at least 6 months?

- ☐ Never
- ☐ Yes, but not now
- ☐ Yes, at present

} → ***If yes, duration of living together*** [ ][ ] years

6.2 How frequently are you exposed to other people's tobacco smoke either at home, workplace or in public places? *(i.e. a minimum of 5 consecutive minutes each time)*

- ☐ Never or almost never
- ☐ Occasionally (<1 time/week)

} → ***Go to Q6.4***

- ☐ 1-2 days/week
- ☐ 3-5 days/week
- ☐ Daily or almost every day

6.3 What is the usual duration of your exposure per week? [ ][ ] Hours

6.4 During past year, how long did you store pesticides at home? [ ][ ] Months

6.5(were6.4a)**Please tell us the duration you lived in 3 most recent houses** (each for at least 1 year)?

- **Present house** [ ][ ] years
- **Previous house** [ ][ ] years
- **The house before previous** [ ][ ] years

6.6(were6.5) **In your present & two previous houses, how often did you cook at home?**

- ☐ Daily
- ☐ Weekly
- ☐ Monthly
- ☐ Never/Rarely → ***Go to Q6.10*** **(wereQ6.8)**
- ☐ No cooking facility → ***Go to Q6.11*** (wereQ6.11)

6.7(were6.6)**In your present & two previous houses, what was the main cooking fuel used?**

- ☐ Gas
- ☐ Coal
- ☐ Wood
- ☐ Eletricity
- ☐ Other

6.8(were6.7)**In your present & two previous houses, what was the main cooking oil used?**

- ☐ Rapeseed
- ☐ Peanut
- ☐ Soybean
- ☐ Lard
- ☐ Other

6.9(were6.7a)**How much time have you spent on cooking so far today?** [ ][ ] minutes

6.10(were6.8)**In your present & two previous houses, did your stove(s) all have a chimney / extractor?**

☐ Yes ☐ Not all stoves ☐ No

6.11(were6.9)**In your present & two previous houses, was your stove always kept under slow burning throughout the day?**

☐ Yes, always ☐ Yes, sometimes ☐ No → if ticked, ***Go to Q6.14***

6.12(were 6.9a) **If yes , types of the fuel most commonly used?**

☐ Smokeless coal ☐ Coal brick / Coalite

☐ Smoky coal ☐ Other

6.13(were 6.9b) **And , the place where stove was usually kept?**

☐ Inside the house ☐ Outside the house

6.14(were6.11)**In winter, did you normally heat your house?**

☐ Yes, ☐ No

6.15 If yes, what was the main heating fuel used?

☐ Central heating ☐ Wood

☐ Gas ☐ Electricity

☐ Coal ☐ Other

6.16(were6.12)**From what year did the inside of your house tend to be coal-smoky in winter?**

☐ Never → if ticked, ***Go to section7***

☐ Ever since childhood

☐ Since the year: _________ year

6.17(were6.13)**In what year did the inside of your house stop being really coal-smoky in winter?**

☐ In the year: _________ year

☐ Still is

Section 7: Personal & family medical history

7.1 How is your current general health status?

7.1.1 Self-rated health status?
- ☐ Excellent
- ☐ Good
- ☐ Fair
- ☐ Poor

7.1.2. Compared to someone of your own age?
- ☐ Better
- ☐ About the same
- ☐ Worse
- ☐ Don't know

7.2 If you were walking on level ground with other healthy people of the same age, would you usually:

7.2.1 Become short of breath?
- ☐ Yes
- ☐ No
- ☐ Disabled

7.2.2 Slow down due to chest discomfort?
- ☐ Yes
- ☐ No
- ☐ Disabled

7.3 During the past 12 months, have you usually had the following symptoms?

7.3.1 Cough frequently?
- ☐ No
- ☐ Yes, for <3 months
- ☐ Yes, for ≥3months

7.3.2 Cough up sputum after getting up in the morning?
- ☐☐ No
- ☐☐ Yes, for <3 months
- ☐☐ Yes, for ≥3 months

7.4 Has a doctor EVER told you that you had had the following disease?

	Diagnosed disease?		Age at first diagnosis	Still on treatment?	
	Yes	No		Yes	No
Diabetes	☐	☐	______	☐	☐
CHD	☐	☐	______	☐	☐
Stroke or TIA	☐	☐	______	☐	☐
Hypertension	☐	☐	______	☐	☐
Rheumatic heart dis.	☐	☐	______	☐	☐
TB	☐	☐	______	☐	☐
Emphysema/bronchitis	☐	☐	______	☐	☐
Asthma	☐	☐	______	☐	☐
Cirrhosis/chronic hepatitis	☐	☐	______	☐	☐
Peptic ulcer	☐	☐	______	☐	☐
Gallstone/gallbladder dis.	☐	☐	______	☐	☐
Kidney disease	☐	☐	______	☐	☐
Fracture	☐	☐	______	☐	☐
Rheumatoid arthritis	☐	☐	______	☐	☐
Psychiatric disorders	☐	☐	______	☐	☐
Neurasthenia	☐	☐	______	☐	☐
Head injury	☐	☐	______	☐	☐
Cancer*	☐	☐	______	☐	☐

For CHD, stroke, and hypertension what is the current medication:

1. Aspirin
2. ACE-I
3. Beta-blocker
4. Statins
5. Diuretics
6. Ca^{++} antagonist

& for diabetes, the above list plus

7. Chlorpropamide or metformin
8. Insulin

***If yes, please indicate the site of cancer** ☐ *(If more than one, choose the one that occurred first)*

0. Lung 1. Esophagus 2. Stomach 3. Liver 4. Intestine 5. Breast 6. Prostate 7. Cervix 8. Other

7.5 **Have many blood transfusions have you ever received?** *(If none, put 0)* ☐☐ times

7.6(were7.5a)**How many times have you ever donated blood for financial payment?**
(If none, put 0) ☐☐ times

7.7(were7.6)**About how often do you have bowel movements each week?**

- ☐ More than once on most days
- ☐ About daily
- ☐ Once every 2-3 days
- ☐ Less than 3 times a week

7.8(were7.7)**How often do your gums bleed when you brush your teeth?**

- ☐ Occasionally, rarely or never
- ☐ Sometimes
- ☐ Always
- ☐ Brush teeth rarely or never

7.9(were7.8)**How many brothers & sisters do you have?***(Including half siblings. If unknown, put#)* ☐☐

7.10(were7.9)**How many children do you have?** *(Including only biological ones)* ☐☐

7.11(were7.9a)**Is your mother still alive?**

- ☐ Yes → If ticked, current age: ☐☐
- ☐ No → If ticked, age at death: ☐☐
- ☐ Unknown

7.12(were7.9b)**Is your father still alive?**

- ☐ Yes → If ticked, current age: ☐☐
- ☐ No → If ticked, age at death: ☐☐
- ☐ Unknown

7.13(were7.10) **Did any of your parents, siblings or children have following diseases?** *(For sibling and children, please record the number with disease)*

	Stroke	Heart attack	Diabetes	Mental disorder	Cancer
**Mother** *(tick box)*	☐	☐	☐	☐	☐
**Father** *(tick box)*	☐	☐	☐	☐	☐
**Siblings** *(inclu. half)*	☐	☐	☐	☐	☐
**Children**	☐	☐	☐	☐	☐

Section 8U: Physical activities (Urban)

8.1 During the past 12 months, how active were you at work?

- ☐ Mainly sedentary (e.g. office worker)
- ☐ Standing occupation (e.g. guard, shop assistant)
- ☐ Manual work (e.g. plumber, carpenter)
- ☐ Heavy manual work (e.g. miner, construction worker)
- ☐ Retired or housewife/husband or unemployed or disabled → ***If ticked, please go to Q8.8***

8.2(were8.1a) **In a typical week, about how many hours did you usually work? _______ hours**

8.3(were8.2) **During the past 12 months, how did you usually get to work?**

- ☐ Mainly walk
- ☐ By motorbike
- ☐ By bicycle
- ☐ By bus/car/ferry/train
- ☐ Mainly stay at home or work near home ↳***If ticked, please go to Q8.8***

8.4(were8.3) **How much time did you spend each day on journey to & from work? _____minutes**

Section 8F: Physical activities (New section for rural farmers)

8.1 During the past 12 months, did your farming work change seasonally?

- ☐ No → ***go to Q8.3***
- ☐ Yes

8.2 During the farming season in the last 12 months:

8.2.1 How many months did it usually last? ☐☐ month

8.2.2 What types of work did it usually involve?

☐ manual ☐ Semi-mechanized ☐ Fully mechanized

8.2.3 How many hours did you usually work each day? ☐☐ hours

8.2.4 Of which , how many hours did you sweat or have a much faster heartbeat? ☐☐ hours

---

8.3 In a typical week, how many hours did you usually work in the field? ☐☐ hours

---

8.4 Apart from agriculture work, did you have any other job?

- ☐ No → ***go to Q8.7***
- ☐ Yes

8.5 How active were you at work with other job?

- ☐ Mainly sedentary
- ☐ Mainly standing
- ☐ Mainly general manual work
- ☐ Mainly heavy manual work

8.6 In a typical week, about how many hours did you work at other job? ☐☐ hours

---

8.7 In a typical day how much time did you usually spend on the journey to and from work on foot or by bicycle? ☐☐☐ minutes

---

Section 8C: Physical activities (Common to both rural farmers and urban)

8.8 During the past 12 months, how often did you do exercise in your leisure time?

- ☐ Never or almost never } → ***If ticked, please go to Q8.11***
- ☐ 1-3 times/month
- ☐ 1-2 times/week
- ☐ 3-5 times/week
- ☐ Daily or almost every day

8.9 What is your main type of exercise? *(tick one box only)*

- ☐ Taichi / Qigong
- ☐ Jogging/aerobic exercise
- ☐ Ball games (basketball, table tennis, etc)
- ☐ Walking
- ☐ Swimming
- ☐ Other (eg. mountain climbing)

8.10 About how many hours per week did you do such exercise in leisure time? ___ hours

---

8.11 In a typical week during the past 12 months, how often did you sweat or have a much faster heartbeat because of heavy physical activities/exercise?

- ☐ Never or almost never } → ***If ticked, please go to Q8.13***
- ☐ <1 time / week
- ☐ 1-2 times/week
- ☐ 3-5 times/week
- ☐ Daily or almost every day

8.12 About how many hours per week did you do such activities? _______ hours

---

8.13 About how many hours per week did you do house work? _______ hours

8.14 About how many hours per week did you watch TV or read? _______hours

---

8.15 During the past 12 months, has your weight changed significantly?

☐ About the same as before ☐ Yes, gained ≥2.5 kg ☐ Yes, lost ≥2.5 kg

---

8.16 Have you tried to reduce weight in the past 12 months? No ☐, Yes ☐

8.17 How much did you weigh when you were at age 25? (If unknown put #) ☐☐☐ jin

---

Section 9: Reproductive history (for women)

9.1 How old were you when you had your first menstrual period? [ ][ ] Year

---

9.2 Have you had your menopause?

- ☐ No
- ☐ Yes, currently
- ☐ Yes, had menopause → If so, age of completion of menopause: [ ][ ] Year

---

9.3 How many times have you ever been pregnant? (if none, put 0. Go to Q9.6) [ ][ ] times

— **Of which,**

Live birth [ ][ ] times → If none, Go to Q9.5 (wereQ9.6)

Still birth [ ][ ] times, Spontaneous abortion [ ][ ] times, Induced abortion [ ][ ] times

---

9.4 Age and length of breastfeeding at each live birth (twins=one birth)?

Live Birth	Age at end of pregnancy	Months of breastfeeding
1st	[ ][ ]	[ ][ ]
2nd	[ ][ ]	[ ][ ]
3rd	[ ][ ]	[ ][ ]
4th	[ ][ ]	[ ][ ]
5th	[ ][ ]	[ ][ ]

9.5(were9.6)**Have you ever used oral contraceptive pills?**

- ☐ Never → ***If ticked, please go to Q9.8*** (were Q9.9)
- ☐ Past use → **if ticked,** age when you last stopped the pill: [ ][ ] Year
- ☐ Current use

9.6(were9.7)**How old were you when you first used oral contraceptives**? [ ][ ] Year

9.7(were9.8)**For how long altogether have you used oral contraceptives?** [ ][ ] Year

---

9.8(were9.9)**Have you had a hysterectomy**?

☐ No , ☐ Yes → If yes, age when you had the operation [ ][ ] Year

---

9.9(were9.10)**Have you had one or both ovaries removed?**

☐ No , ☐ Yes → If yes, age when you had the most recent operation [ ][ ] Year

---

9.10(were9.11) **Have you ever had surgery to remove a breast lump?**

☐ No, ☐ Yes → If yes, age when you most recently had the operation [ ][ ] Year

---

Section 10: Sleeping, mood & mental situation

10.1 In general, how satisfied are you with your life?

- ☐ Very satisfied
- ☐ Satisfied
- ☐ Neither satisfied nor dissatisfied
- ☐ Unsatisfied
- ☐ Very unsatisfied

10.2 Over the past two years have you had any of the following major events in your life?

Yes	No		Yes	No	
☐	☐	Marital separation/divorce	☐	☐	Major injury or traffic accident
☐	☐	Loss of job/retirement	☐	☐	Death /major illness of spouse
☐	☐	Business bankrupt	☐	☐	Death/major illness of other close family member
☐	☐	Violence	☐	☐	Major natural disaster (e.g. flood & drought)
☐	☐	Major conflict within family	☐	☐	Loss of income / living on debt

---

10.3 During the past month, did you have any of the following for ≥3 days each week?

Yes	No	
☐	☐	Taking >30 minutes to fall asleep after going to bed or waking up in the middle of the night
☐	☐	Waking up early and not being able to go back to sleep
☐	☐	Needing to take medicine (including herbal or sleeping pills) at least once a week to help sleep
☐	☐	Having difficulty staying alert while at work, eating or meeting people during daytime

---

10.4 Do you usually take a daytime nap? ☐ Yes usually, ☐ Yes ,but only in summer , ☐ No

10.5(were10.4a) **Do you snore during sleep?** ☐ Yes, Frequently, ☐ Yes, Sometimes, ☐ No / Don't know

10.6(were10.5)**How many hours do you typically sleep per day (incl. naps)?** ☐☐ Hours

---

10.7(were10.6)**During the past 12 months, have you had following situations for 2 or more weeks?**
(If answer yes to any of the questions, complete CIDI-A)

Yes	No	
☐	☐	Feeling much more sad, or depressed than usual
☐	☐	Loss of interest in most things like hobbies or activities that usually give you pleasure
☐	☐	Felt so hopeless that you had no appetite to eat even your favourite food
☐	☐	Feeling worthless and useless, everything went wrong was your fault and life is very difficult that there was no way out

---

10.8(were10.7) **During the past 12 months, have you experienced the following situations?**

Yes	No	
☐	☐	Having a period lasting one month or longer when most of time you felt worried, tense, or anxious and it interfered your life *(if yes, complete CIDI-B)*
☐	☐	Having a pain or discomfort in your body lasting ≥3 months that interfered with your life
☐	☐	Having had a spell or an attack when all of sudden felt frightened, anxious, or very uneasy
☐	☐	Having had inexplicable strong fear in situations such as closed space (cave, elevator, airplane etc), in the crowds or public such that you would avoid such situations

---

Section 11: Physical examination

11.1 Standing height (without shoes) …………………… [| | | .] m

11.2 Sitting height …………………………………… [| | | .] cm

11.3 Waist ………………………………………………… [| | | .] cm

11.4 Hip ………………………………………………… [| | | .] cm

11.5 Weight (without shoes, but in light clothing) ………… [| | | .] Kg

11.6 BMI ………………………………………………… [| | .] Kg/m^2

11.7 Impedance ………………………………………… [| |] Ω

11.8 Fat % (with one decimal point) ……………………… [| .]

Staff code [| |]

11.9 Did you take any drugs to lower blood pressure in the last 2 days? ☐ Yes ☐ No

11.10 Blood pressure & heart rate (to be measured after 5 minutes in the seated position)

	First	Second	
**SBP**	[		]	[		]	mmHg
**DBP**	[		]	[		]	mmHg
**Heart rate**	[		]	[		]	bpm

11.11(were11.10a)**Hours since last ate anything (ignore any drinks)? _______ hours**

11.12(were11.11)**Blood sample collected:** Yes (, . Failed (

Staff code [| |]

11.13 (were11. 12) **Lung function & CO levels:**

	First	Second	
**CO**	[		]	[		]	
**%COHB**	[		.		]	[		.		]	%
**FEV1**	[	.		]	[	.		]	Liter
**FVC**	[	.		]	[	.		]	Liter

Staff code [| |]

11.14(were11.13)Assessment of subject's cooperation and the reliability of data collected?

Assessment of subject's cooperation?

☐ Good
☐ Fair
☐ Poor

11.15 Assessment of the reliability of the information collected?

☐ Good
☐ Fair
☐ Poor

Date of interview ______Year______Month____Day, **Signature of interviewer___________**

11 December 2013

China Kadoorie Biobank

【2nd Resurvey Questionnaire】

The items in red are changes from the first resurvey.

Version 2.2, CKB/ICC/2013

Section 1: Background information

1.1 Resurvey ID: | K | | 2 | | | | | | | | | | | | | **Baseline ID:** | K | | 0 | | | | | | | | | | | | |

1.2 Name: ______________, **Sex:** Male ☐ Female ☐ , **Name of spouse:**____________

1.3 Date of birth: ☐☐☐☐ Year ☐☐ Month ☐☐ Day

1.4 National ID number (if no, put #) ☐☐☐☐☐☐☐☐☐☐☐☐☐☐☐☐☐☐

1.5 Home address: _____ Province _____ City_____ District/County ______ Street/Village

Home telephone: Not available ☐, Yes: ☐☐☐☐☐ – ☐☐☐☐☐☐☐☐☐☐

Mobile telephone: Not available ☐, Yes: ☐☐☐☐☐☐☐☐☐☐☐☐☐☐☐

1.5.1 Were you born in this province [i.e. where the interview is taking place] (to the best of your knowledge)?

- ☐ Yes
- ☐ No →***If ticked, Go to <u>1.5.1a</u>***
- ☐ Don't know

1.5.1a Where were you born?

Drop down list of 31 provinces with "Other" and "Don't know" at the end **(see Appendix 1)**

1.5.2 Were any of your parents or grandparents born outside the province where you were born?

- ☐ Yes
- ☐ No
- ☐ Don't know

1.5.3 Are you Han Chinese?

- ☐ Yes
- ☐ Partly (i.e. Han-mixed)
- ☐ No
- ☐ Don't know

1.5.4 Are any of your parents and/or grandparents non-Han or mixed ethnic group?

- ☐ Yes
- ☐ No
- ☐ Don't know

1.5.5 Do you have a religion?

- ☐ Yes, →If ticked, specify type (**<u>Q1.5.5a</u>**): ☐ Buddhist, ☐ Christian, ☐ Muslim, ☐Other
- ☐ Do not wish to say
- ☐ No

1.6 What is the highest level of school education you ever received?

- ☐ No formal school
- ☐ Primary School
- ☐ Middle School
- ☐ High School / Technical School
- ☐ College
- ☐ University

1.7 What is your current occupation?

- ☐ Agriculture & related workers
- ☐ Factory worker
- ☐ Administrator / manager
- ☐ Professional / technical
- ☐ Sales & service workers
- ☐ Retired. ***If "YES", Go to Q1.7.1 & Q1.7.2***
- ☐ House wife / husband
- ☐ Self-employed
- ☐ Unemployed
- ☐ Other or not stated

1.7.1 What was your last occupation before you retired?

- ☐ Agriculture & related workers
- ☐ Factory worker
- ☐ Administrator / manager
- ☐ Professional / technical
- ☐ Sales & service workers
- ☐ House wife / husband
- ☐ Self-employed
- ☐ Unemployed
- ☐ Other or not stated

1.7.2 Why did you retire?

- ☐ Reaching retirement age
- ☐ Health related (excluding injury)
- ☐ Other

1.8 How many people live together as a family in the household? ☐☐ persons

---

1.9 What is your current marital status

- ☐ Married
- ☐ Widowed
- ☐ Separated / divorced
- ☐ Never married

1.10 What is the total income last year in your household?

- ☐ <2,500 yuan
- ☐ 2,500-4,999 yuan
- ☐ 5,000-9,999 yuan
- ☐ 10,000-19,999 yuan
- ☐ 20,000-34,999 yuan
- ☐ 35,000-49,999 yuan
- ☐ 50,000-74,999 yuan
- ☐ 75,000-99,999 yuan
- ☐ ≥100,000 yuan

1.11 Do you have any of the following items in your household?

Yes	No	
☐	☐	Health care cover (for yourself only)
☐	☐	Own house / apartment
☐	☐	Tap water in your own house *(Note: it was option 7 in last survey)*

☐ ☐ Toilet for private use
☐ ☐ Telephone or mobile phone
☐ ☐ Car *(Note: used to be in the same question as Motorbike)*
☐ ☐ Motorbike / other motor vehicle
☐ ☐ Computer (incl. laptop, tablet, iPad)
☐ ☐ Web access
☐ ☐ Email/QQ (for yourself only)
☐ ☐ Holiday during last five years

Section 2: Tea & coffee drinking

2.1 During the past 12 months, how often did you drink any tea?

- ☐ Never or almost never
- ☐ Only occasionally
- ☐ Only at certain seasons
- ☐ Every month but less than weekly
- ☐ Usually at least once a week → ***If ticked, Go to <u>Q2.3</u>***

2.2 In the past, did you ever have a period of at least 1 year during which you usually drank tea at least once a week?

- ☐ Yes, → if so, how long ago did it end (**<u>Q2.2a</u>**)? ☐☐ Years } ***Go to <u>Q2.11</u>***
- ☐ No

2.3 During the past 12 months, on how many days did you drink tea in a typical week?

- ☐ 1-2 days/week
- ☐ 3-5 days/week
- ☐ Daily or almost every day

2.4 At about what age did you start drinking tea in most weeks? ☐☐ Years

---

2.5 On days when you drink tea, how many cups do you usually drink? (choose one only)

	cups/day
Green /Jasmine tea	☐☐
Oolong tea	☐☐
Black tea	☐☐
Other tea	☐☐

2.6 How often do you change tea leaves during a day? ☐☐ times

---

2.7 About how much tea leaves do you usually add each time? ☐☐ grams

---

2.8 What strength of tea do you usually prefer to drink?

- ☐ Weak
- ☐ Moderate
- ☐ Strong

2.9 At about what temperature do you usually drink your tea?

- ☐ Room temperature / warm
- ☐ Hot
- ☐ Burning hot

2.10 Has your tea consumption changed significantly compared with that some years ago? ☐ About the same as before, ☐ Has increased a lot, ☐ Has decreased a lot

---

2.11 During the past 12 months, how often did you drink any coffee?

- ☐ Never or almost never
- ☐ Only occasionally
- ☐ Every month but less than weekly
- ☐ Usually at least once a week

Section 3: Alcohol consumption

3.1 During the past 12 months, how often did you drink any alcohol?

- ☐ Never or almost never
- ☐ Only occasionally
- ☐ Only at certain seasons
- ☐ Every month but less than weekly
- ☐ Usually at least once a week → ***If ticked, Go to Q3.3***

3.2 In the past, did you ever have a period of at least 1 year, during which you usually drank some alcohol at least once a week?

- ☐ Yes → If so, how long ago did it end (**Q3.2a**)? ☐☐ Years → ***Go to Q3.2b***
- ☐ No → ***Go to section 4***

3.2b What was your main reason for stopping?

- ☐ Physical illness that you already had
- ☐ Health concerns (about future illness)
- ☐ Money
- ☐ Family against
- ☐ Doctor's advice
- ☐ Other

→ ***Go to section 4***

3.3 During the past 12 months, on how many days did you drink alcohol in a typical week?

- ☐ 1-2 days/week
- ☐ 3-5 days/week
- ☐ Daily or almost every day

3.4 At about what age did you start drinking some alcohol in most weeks? ☐☐ **years**

3.5 On three different situations, what kind(s) of alcoholic drinks you choose and how much you drink in a day?

(If used to drink more than one kind on a single occasion, can choose up to 3 types of alcohol for all occasions; fill in other fields with 0)

Alcohol type	On a typical day	On a special day when you drink a lot	Last time when you drank
Beer (large)	☐☐ bottle	☐☐ bottle	☐☐ bottle
Rice Wine	☐☐ liang	☐☐ liang	☐☐ liang
Wine	☐☐ liang	☐☐ liang	☐☐ liang
Spirit (⩾40% alcohol)	☐☐ liang	☐☐ liang	☐☐ liang
Spirit (<40% alcohol)	☐☐ liang	☐☐ liang	☐☐ liang

3.6 On a typical day when you drink alcohol, when do you usually take the drink?

- ☐ Usually drink with the meal
- ☐ Usually drink between or after the meals

☐ No regular pattern

3.7 After drinking alcohol, do you usually experience hot flushes or dizziness?

☐ Yes, soon after first mouthful →***If ticked, Go to Q3.8***

☐ Yes, after drinking small amount of alcohol →***If ticked, Go to Q3.8***

☐ Yes, but only after drinking large amount of alcohol

☐ No

3.7.1 In the first one or two years when you started drinking regularly, did you experience hot flushes or dizziness?

☐ Yes, soon after first mouthful

☐ Yes, after drinking small amount of alcohol

☐ Yes, but only after drinking large amount of alcohol

☐ No

3.8 During the past month, how often have you drunk alcohol in the morning?

☐ Never

☐ <1 day/week

☐ A few days a week

☐ Daily or almost daily

3.9 During the past month, have you ever had the following experiences?

Yes	No	
☐	☐	Unable to work or to do anything because of drinking
☐	☐	Felt depressed, angry or couldn't control yourself after drinking
☐	☐	Could not keep away from drinking
☐	☐	Had shakes when you stopped drinking

3.10 Has your alcohol consumption changed significantly compared with that some years ago?

☐ About the same as before

☐ Has increased a lot

☐ Has decreased a lot

3.11 Have you drunk any alcohol today? (previously Q3.1) ☐ Yes, ☐ No

Section 4: Smoking history

4.1 Have you smoked any tobacco today? ☐ **Yes ,** ☐ **No,** → if yes, **(Q4.1a)** how many: __ total, & **(Q4.1b)** how many: ___in last hour

---

4.2 How often do you smoke tobacco now?

- ☐ Do not smoke now
- ☐ Only occasionally

} → ***If ticked, Go to Q4.3***

- ☐ Yes, on most days
- ☐ Yes, daily or almost every day

4.2.1 How soon after waking in the morning do you usually have your first smoke?

- ☐ ≤5 minutes
- ☐ 6-30 minutes
- ☐ 31-60 minutes
- ☐ >60 minutes

After completing Q4.2.1, Go to Q4.7

4.3 In the past, how frequently did you smoke?

- ☐ Did not smoke
- ☐ Smoked only occasionally
- ☐ Smoked on most days
- ☐ Smoked daily or almost every day

} → ***If ticked, Go to Q4.5***

4.4 In your life time, have you smoked a total of at least 100 cigarettes or equivalent?

- ☐ Yes
- ☐ No

} → ***Go to Section 5***

4.5 How many years ago did you last stop smoking regularly? ☐☐ Years ☐☐ Months

---

4.6 What was your main reason for stopping?

- ☐ Physical illness that you already had
- ☐ Health concerns (about future illness)
- ☐ Money
- ☐ Family against
- ☐ Doctor's advice
- ☐ Other

4.7 At about what age did you first start smoking on most days? ☐☐ Years

---

4.8 What tobacco did you use when you first started smoking on most days?

Mainly cigarette ☐, Mainly non-cigarette ☐, Mixed types ☐

→ **Q4.8a** From the time you first started until now or first stopped, did you always smoke some cigarettes on most days, never having a month or more without them? Yes ☐, No ☐

4.9 What kind(s) of tobacco do you usually smoke (or did you smoke before giving up) and how much?

Filter cigarettes (factory) .... [|] number/day

Non-filter cigarettes (factory)...................................... ..... [|] number/day

Hand-rolled cigarettes .... [|] liang/month

Pipe or water pipe .... [|] liang/month

Cigars .... [|] number/day

4.10 How deeply do (or did) you usually inhale the smoke?

☐ Mouth only

☐ Throat

☐ Lung → If ticked, then **Q4.10a**: have you nearly always inhaled a lot of smoke into your lung when smoking?

Yes ☐, No ☐

If ex-smoker (ie, answered Q4.5), move to Q4.12c

4.11 Has your tobacco consumption changed significantly compared with that some years ago?

☐ About the same as before, ☐ Has increased a lot, ☐ Has decreased a lot

---

4.12 Have you ever tried to quit smoking (without smoking for at least one week)?

☐ Yes, ☐ No; →***If "No", then Go to Section 5:***

4.12a How many years ago did you last try to quit? [|] Years [|] Months

4.12b How long did it last? [|] Years [|] Months

4.12c Have you ever used the following methods to assist with smoking cessation?

Yes	No	
☐	☐	Accupuncture
☐	☐	Nicotine gum/patch
☐	☐	Bupropion
☐	☐	Chanpix
☐	☐	Smoking cessation clinics
☐	☐	Special support group
☐	☐	Quitline
☐	☐	Exercise routine
☐	☐	Any other methods

Section 5: Diet

5.1 During the past 12 months, about how often did you consume the following foods or drinks?

**Staple foods**	**Daily**	**4-6 days Per week**	**1-3 days Per week**	**Monthly**	**Never or rarely**	**On the day when you consume the foods or drinks, about how much do you consume (Q5.1a)?**
Rice	☐	☐	☐	☐	☐	_____Liang
Wheat	☐	☐	☐	☐	☐	_____Liang
Other staple foods (corn, millet etc.)	☐	☐	☐	☐	☐	_____Liang
**Animal foods**						
Meat	☐	☐	☐	☐	☐	_____Liang
Poultry	☐	☐	☐	☐	☐	_____Liang
Fish/sea food	☐	☐	☐	☐	☐	_____Liang
Fresh eggs	☐	☐	☐	☐	☐	_____Ge
**Vegetables**						
Fresh vegetables	☐	☐	☐	☐	☐	_____Liang
Soya products (excluding liquids)	☐	☐	☐	☐	☐	_____Liang
Dried vegetables (mushrooms, agaric, etc)	☐	☐	☐	☐	☐	_____Liang
Salted vegetables	☐	☐	☐	☐	☐	_____Liang
Pickled vegetable (sour taste)	☐	☐	☐	☐	☐	_____Liang
**Other foods**						
Yoghurt	☐	☐	☐	☐	☐	_____Liang
Other dairy foods (milk powder, cheese etc)	☐	☐	☐	☐	☐	_____gram
Fresh fruits	☐	☐	☐	☐	☐	_____Portions
**Drinks**						
Soymilk	☐	☐	☐	☐	☐	_____ml
Milk	☐	☐	☐	☐	☐	_____ml
Pure fruit/vegetable juice	☐	☐	☐	☐	☐	_____ml
Carbonated soft drinks	☐	☐	☐	☐	☐	_____ml
Other cold soft drinks	☐	☐	☐	☐	☐	_____ml

5.2 During the past 12 months, about how often did you do the following things?

	Daily	4-6 days per week	1-3 days per week	Monthly	Never or rarely
Snacking (including late-night snacks)	☐	☐	☐	☐	☐
Skipping breakfast	☐	☐	☐	☐	☐
Eating in restaurants, street food stalls etc.	☐	☐	☐	☐	☐
Eating deep fried foods	☐	☐	☐	☐	☐
Eating Western-type fast foods (eg pizza/burgers)	☐	☐	☐	☐	☐

5.3 What is your preferred saltiness for your dishes compared with your friends or colleagues?

☐ Very light
☐ About average
☐ Very salty

5.4 About how many days on average would the following cooking ingredients usually last in your household? (if none/unknown enter #)

a) A bag (500 gram) of salt: ☐☐☐ days
b) A bottle (500 ml) of soy sauce: ☐☐☐ days
c) A bottle (1 liter) of cooking oil: ☐☐☐ days

5.5 During the past 12 months, have you taken the following supplements regularly?

Yes	No	
☐	☐	Fish oil/cod liver oil
☐	☐	Vitamins
☐	☐	Calcium/iron/zinc
☐	☐	Ginseng and related products
☐	☐	Traditional Chinese medicine
☐	☐	Other herbal health supplements

5.6 Have you ever experienced any severe food shortage? ☐ Yes, ☐ No→ **if No, *Go to Q5.9***

5.7 What year was the worst food shortage you experienced? ☐☐ years

5.8 During the most severe food shortage have you experienced following situations?

Yes	No	Don't know	
☐	☐	☐	Lose weight? → If "Yes", then **Q5.8a**: by about how much ____ *jin*
☐	☐	☐	Develop specific diseases related to food shortage?
☐	☐	☐	Death of family member or close friend/neighbour during food shortage?

5.9 Did your mother experience any severe food shortage when expecting you?

☐ Yes, ☐ No, ☐ don't know

5.10 How many years have you had a refrigerator in your home? ☐☐ Years

5.11 During the past month, about how often did you eat hot spicy food?

- ☐ Never or almost never
- ☐ Only occasionally

} → ***Go to Section 6***

- ☐ 1-2 days/week
- ☐ 3-5 days/week
- ☐ Daily or almost every day

5.12 At what age did you start to eat spicy food at least once a week? ☐☐ Years

5.13 What strength of spicy food do you usually prefer to eat?

☐ Weak, ☐ Moderate, ☐ Strong

5.14 On day when you eat spicy food, what are the main sources of spice usually used?

Yes	No	
☐	☐	Chili sauce
☐	☐	Chili oil
☐	☐	Dried chili pepper
☐	☐	Fresh chili pepper
☐	☐	Other or don’t know

Section 6: Passive smoking & air pollution

6.1 Have you ever lived with a smoker in the same house for at least 6 months?

☐ Never

☐ Yes, but not now

☐ Yes, at present

} → ***If yes, Q6.1a duration of living together*** [|] Years

*If 1st box is ticked, then **Go to Q6.3.** If 2nd box is ticked, then **Go to Q6.3** after answering duration*

6.2 During the past 12 months, how frequently have you been exposed to tobacco smoke from a family member at home or someone you shared a room with? *(i.e. a minimum of 5 consecutive minutes each time)*

☐ Occasionally (<1 day / week) → ***If ticked, Go to Q6.3***

☐ 1-2 days/week

☐ 3-5 days/week

☐ Daily or almost every day

6.2.1 What is the usual duration of your exposure per week? [|] Hours

---

6.3 During the past 12 months, about how frequently have you been exposed to other people's tobacco smoke in workplace or public places? *(i.e. a minimum of 5 consecutive minutes each time)*

☐ Never or almost never

☐ Occasionally (<1 time/week)

} → ***If ticked, Go to Q6.4***

☐ 1-2 days/week

☐ 3-5 days/week

☐ Daily or almost every day

6.3.1 What is the usual duration of your exposure per week? [|] Hours

---

6.4 During past year, how long did you store pesticides at home? [|] Months

---

6.5 Please tell us the duration you lived in 3 most recent houses (each for at least 1 year)?

Present house [|] years

Previous house [|] years

The house before previous [|] years

6.6 How far are your present & previous houses from any main/busy road with traffic?

Present house: ☐ <50m, ☐ 50-100m, ☐ 100-200m, ☐ >200m

Previous house1: ☐ <50m, ☐ 50-100m, ☐ 100-200m, ☐ >200m (skip if #)

Previous house2: ☐ <50m, ☐ 50-100m, ☐ 100-200m, ☐ >200m (skip if #)

6.7 Are your present & previous houses within 10 kilometres of heavily air-polluted (with smoke/gas/dust) industry/industrial zone?

Present house: ☐ Yes ☐ No

Previous house1: ☐ Yes ☐ No (skip if Previous house1 is #)
Previous house2: ☐ Yes ☐ No (skip if Previous house2 is #)

6.8 During the past 12 months, how often did you cook at home?

☐ Daily or almost every day
☐ A few times a week
☐ A few times a month → ***If ticked, Go to Q6.9***
☐ Never or rarely
☐ No cooking facility → ***If ticked, Go to Q6.11***

6.8.1 How much time on average do you spend in front of the fire/stove while cooking per week? ________ Hours

6.8.2 At about what age did you start cooking regularly at home? |__|__| Years

6.8.3 What is the main cooking oil used now?

☐ Rapeseed ☐ Lard
☐ Peanut ☐ Other
☐ Soybean

6.9 In your household, what is the main cooking fuel used now?

☐ Gas ☐ Electricity
☐ Coal →***If ticked, Go to Q6.9a*** ☐ Other
☐ Wood

6.9a Please specify the main type of coals used?

☐ Smokeless coal ☐ Coal brick / Coalite (smokeless)
☐ Smoky coal ☐ Other

6.10 In your household, do all stoves have a chimney / extractor?

☐ Yes ☐ Not all stoves ☐ No

6.10.1 Apart from cooking, is your stove always kept under slow burning throughout the day?

☐ Yes, always ☐ Yes, sometimes ☐ No → ***If ticked, Go to Q6.11***

6.10.2 Where is the stove usually kept?

☐ Inside the house ☐ Outside the house

6.11 In winter, do you normally heat your house?

☐ Yes, ☐ No → ***If No, Go to Q6.12***

6.11.1 What is the main heating fuel?

☐ Central heating ☐ Wood
☐ Gas ☐ Electricity
☐ Coal →***If ticked, Go to Q6.11.2*** ☐ Other

6.11.2 Please specify the main type of coals used:

☐ Smokeless coal ☐ Coal brick / Coalite (smokeless)

☐ Smoky coal ☐ Other

6.11.3 When you heat the house in winter, do you still need to wear plenty of warm clothes at home? ☐ Yes, ☐ No

---

6.12 From what year did the inside of your house tend to be coal-smoky in winter?

☐ Never → ***If ticked, Go to Q6.13***

☐ Ever since childhood

☐ Since the year: _________ year

6.12.1 In what year did the inside of your house stop being really coal-smoky in winter?

☐ In the year: _________ year

☐ Still is

6.13 During your working life, have you ever been exposed to any of the following for at least 6 consecutive months?

Gas/Vapour/Fume/Mist ☐ Yes, ☐ No;→if Yes, then **Q6.13a** total years of exposure? ___years

Dust (eg. silica/coal/cotton) ☐ Yes, ☐ No;→if Yes, then **Q6.13b** total years of exposure? ___years

Fibres (eg, asbestos/textile) ☐ Yes, ☐ No;→if Yes, then **Q6.13c** total years of exposure? ___years

---

Section 7: Personal & family medical history

7.1 How is your current general health status?

7.1.1 Self-rated health status?

- ☐ Excellent
- ☐ Good
- ☐ Fair
- ☐ Poor

7.1.2. Compared to someone of your own age?

- ☐ Better
- ☐ About the same
- ☐ Worse
- ☐ Don't know

7.2 If you were walking on level ground with other healthy people of the same age, would you usually:

7.2.1 Become short of breath?

- ☐ Yes
- ☐ No
- ☐ Disabled

7.2.2 Slow down due to chest discomfort?

- ☐ Yes
- ☐ No
- ☐ Disabled

7.3 During the past 12 months, have you usually had the following symptoms?

7.3.1 Cough frequently?

- ☐ No
- ☐ Yes, for <3 months
- ☐ Yes, for ≥3months

7.3.2 Cough up sputum after getting up in the morning?

- ☐ No
- ☐ Yes, for <3 months
- ☐ Yes, for ≥3 months→ If yes, **Q7.3.2a** for how long ___ years

7.3.3 Wheeze or whistle in the chest?

- ☐ No
- ☐ Yes
- ☐ Yes, but only when having a cold or viral infection

7.4 Has a doctor EVER told you that you had had the following disease?

	Diagnosed disease?		Q7.4a	Q7.4b**		Q7.4c		Q7.4d
	Yes	No	Age of first diagnosis	Still on Treatment		Hospitalized? Yes	No	If yes, date of last hospitalisation
Diabetes	☐	☐	________	☐	☐	☐	☐	________
Acute MI	☐	☐	________	☐	☐	☐	☐	________
Angina	☐	☐	________	☐	☐	☐	☐	________
Other IHD	☐	☐	________	☐	☐	☐	☐	________
Stroke or TIA	☐	☐	________	☐	☐	☐	☐	________
Hypertension	☐	☐	________	☐	☐	☐	☐	________
Pulmonary heart dis#	☐	☐	________	☐	☐	☐	☐	________
Rheumatic heart dis.	☐	☐	________	☐	☐	☐	☐	________
TB	☐	☐	________	☐	☐	☐	☐	________
Emphysema#	☐	☐	________	☐	☐	☐	☐	________
Chronic bronchitis#	☐	☐	________	☐	☐	☐	☐	________
COPD#	☐	☐	________	☐	☐	☐	☐	________

Asthma	☐	☐	________	☐	☐	☐	☐	________
Cirrhosis/chronic hepatitis	☐	☐	________	☐	☐	☐	☐	________
Peptic ulcer	☐	☐	________	☐	☐	☐	☐	________
Gallstone/gallbladder dis.	☐	☐	________	☐	☐	☐	☐	________
Kidney disease	☐	☐	________	☐	☐	☐	☐	________
Osteroporosis	☐	☐	________	☐	☐	☐	☐	________
Fracture	☐	☐	________	☐	☐	☐	☐	________
Rheumatoid arthritis	☐	☐	________	☐	☐	☐	☐	________
Depression†	☐	☐	________	☐	☐	☐	☐	________
Anxiety‡	☐	☐	________	☐	☐	☐	☐	________
Neurasthenia	☐	☐	________	☐	☐	☐	☐	________
Other psychiatric disorders	☐	☐	________	☐	☐	☐	☐	________
Head injury	☐	☐	________	☐	☐	☐	☐	________
Cancer*	☐	☐	________	☐	☐	☐	☐	________

***If yes, Q7.4e please indicate the site of cancer** ☐ *(If more than one, choose the first one)*

1. Lung 2. Esophagus 3. Stomach 4. Liver 5. Intestine 6. Breast 7. Prostate 8. Cervix 9. Other

****Note: See Appendix 2 for drug list related to diabetes, CVD (acute MI, angina, other IHD, stroke/TIA) and hypertension.**

† If answered "yes", then go to CIDI-A questionnaire

‡ If answered "yes", then go to CIDI-B questionnaire

7.5 Link to COPD questionnaire: if "Yes" for any of the 4 diagnosed conditions marked with #, or ≥2 years in Q7.3.2a (Appendix 3)

7.6 During the past 12 months, how many times have you visited hospital as an outpatient for any reason? *(If none, put 0)*

times ☐☐

7.7 During the past 12 months, how many times have you been hospitalized overnight for any reason? *(If none, put 0)*

☐☐ times

7.8 How many blood transfusions have you ever received? *(If none, put 0)* ☐☐ times

7.9 How many times have you ever donated blood for financial payment?

(If none, put 0) ☐☐ times

7.10 About how often do you have bowel movements each week?

- ☐ More than once on most days
- ☐ About daily
- ☐ Every other day
- ☐ Less than 3 times a week

7.11 How often do your gums bleed when you brush your teeth?

- ☐ Occasionally, rarely or never
- ☐ Sometimes
- ☐ Always
- ☐ Brush teeth rarely or never
- ☐ Have false teeth

7.12 How many brothers & sisters do you have? *(Including half siblings. If unknown, put#)* ☐☐

7.13 How many children do you have? *(Including only biological ones)* ☐☐

7.14 Is your mother still alive?

- ☐ Yes → If ticked, **Q7.14a** current age: ☐☐ Years
- ☐ No → If ticked, **Q7.14b** age at death: ☐☐ Years
- ☐ Unknown

7.15 Is your father still alive?

- ☐ Yes → If ticked, **Q7.15a** current age: ☐☐ Years
- ☐ No → If ticked, **Q7.15b** age at death: ☐☐ Years
- ☐ Unknown

7.16 Did any of your parents, siblings or children have following diseases? *(For sibling and children, please record the number with disease***)**

	Mother	Father	Siblings (inclu.half)	Children
Stroke	☐ Yes; ☐ No	☐ Yes; ☐ No	☐☐ person	☐☐ person
Heart attack	☐ Yes; ☐ No	☐ Yes; ☐ No	☐☐ person	☐☐ person
Diabetes	☐ Yes; ☐ No	☐ Yes; ☐ No	☐☐ person	☐☐ person
Depression	☐ Yes; ☐ No	☐ Yes; ☐ No	☐☐ person	☐☐ person
Cancer	☐ Yes; ☐ No	☐ Yes; ☐ No	☐☐ person	☐☐ person
COPD/Bronchitis Emphysema/PHD	☐ Yes; ☐ No	☐ Yes; ☐ No	☐☐ person	☐☐ person

7.17 Do you know your birth weight?

- ☐ Yes, → If ticked, **Q7.17a** what was your weight: ☐☐ *jin (rounded to 0.1 jin)*
- ☐ No

7.18 Were you born prematurely? ☐ Yes, ☐ No, ☐ Not sure

7.19 Were you born through Caesarean Section?

- ☐ Yes
- ☐ No
- ☐ Not sure

7.20 Did you suffer any birth-related trauma when born?

- ☐ Yes
- ☐ No
- ☐ Not sure

Section 8U: Physical activities (Non-Agriculture & related workers)

8.1 During the past 12 months, how active were you at work?

- ☐ Mainly sedentary (e.g. office worker)
- ☐ Standing occupation (e.g. guard, shop assistant)
- ☐ Manual work (e.g. plumber, carpenter)
- ☐ Heavy manual work (e.g. miner, construction worker)
- ☐ Retired or housewife/husband or unemployed or disabled → ***If ticked, Go to Q8.9 (was Q8.8)***

8.2 In a typical week, about how many days did you usually work? ____days

8.2a On days when you work, on average how many hours do you work? _____ hours

---

8.3 During the past 12 months, how did you usually get to work?

- ☐ Mainly walk
- ☐ By motorbike/mopad
- ☐ By bicycle
- ☐ By bus/ferry/train
- ☐ By car/taxi
- ☐ Mainly stay at home or work near home
  ↳ ***If ticked, Go to Q8.9 (was Q8.8)***

8.4 How much time did you spend each day on commuting? ____minutes

---

Section 8F: Physical activities (Agriculture & related workers)

8.1 During the past 12 months, did your farming work change seasonally?

- ☐ No → ***If ticked, Go to Q8.3***
- ☐ Yes

8.2 Please specify your activities during the farming season in the last 12 months:

8.2.1 How many months did it usually last? ☐☐ month

8.2.2 What types of work did it usually involve?

☐ Manual ☐ Semi-mechanized ☐ Fully mechanized

8.2.3 How many hours did you usually work each day? ☐☐ hours

8.2.4 Of which, how many hours did you sweat or have a much faster heartbeat? ☐☐ hours

---

8.3 In a typical week (of non-farming season), how many hours did you usually work in the field? ☐☐ hours

---

8.4 Apart from agriculture work, did you have any other job?

- ☐ No → ***If ticked, Go to Q8.7***
- ☐ Yes

8.5 How active were you at work with other job?

☐ Mainly sedentary
☐ Mainly standing
☐ Mainly general manual work
☐ Mainly heavy manual work

8.6 In a typical week, about how many days did you usually work at other job?
____days

8.6a On days when you work at your other job, on average how many hours do you work? _____ hours

---

8.7 During the past 12 months, how did you usually get to work?

☐ Mainly walk
☐ By motorbike
☐ By bicycle
☐ By bus/ferry/train
☐ By car/taxi
☐ Mainly stay at home or work near home
↳ ***If ticked, Go to Q8.9 (was 8.8)***

8.8 How much time in total did you usually spend each day on commuting?
☐☐ minutes

Section 8C: Physical activities (Common to all participants)

8.9 During the past 12 months, how often did you do exercise in your leisure time?

☐ Never or almost never
☐ 1-3 times/month
} → ***If ticked, Go to Q8.12 (was 8.11)***
☐ 1-2 times/week
☐ 3-5 times/week
☐ Daily or almost every day

8.10 What is your main type of leisure exercise? *(tick one box only)*

☐ Taichi / Qigong
☐ Jogging/aerobic dancing
☐ Ball games (basketball, table tennis, etc)
☐ Walking
☐ Swimming
☐ Other (eg. hill walking, mountain climbing, rope jumping, kicking shuttlecock)

8.11 About how many hours per week did you do such exercise in total in leisure time?
______ hours

8.12 In a typical week during the past 12 months, how often did you sweat or have a much faster heartbeat because of heavy physical activities/exercise?

☐ Never or almost never
☐ <1 time / week
} → ***If ticked, Go to Q8.14 (was 8.13)***
☐ 1-2 times/week
☐ 3-5 times/week
☐ Daily or almost every day

8.13 About how many hours per week did you do such activities? ______ **hours**

8.14 About how many hours per day did you do house work? ______ **hours**

8.15 While not working, on average about how many hours per day did you spend on sitting activities, including watching TV, reading etc? ___hours/day

8.16 During the past 12 months, about how often did you do the following sitting activities?

	Daily	4-6 days per week	1-3 days per week	Monthly	Never or rarely	On the day you do that activity, how long do you spent on it? (Q8.16a)
Watching TV/DVD	☐	☐	☐	☐	☐	_______ hours/day
Reading books / newspapers	☐	☐	☐	☐	☐	_______ hours/day
Eating, chatting or socializing	☐	☐	☐	☐	☐	_______ hours/day
Playing cards/mahjong/board games	☐	☐	☐	☐	☐	_______ hours/day
Doing household paper work/writing /internet	☐	☐	☐	☐	☐	_______ hours/day

8.17 During the past 12 months, has your weight changed significantly?

☐ About the same as before ☐ Yes, gained ≥5 *jin* ☐ Yes, lost ≥5 *jin*

8.18 Have you tried to reduce weight in the past 12 months? No ☐, Yes ☐

8.19 How much did you weigh when you were at age 25? (If unknown put #) ☐☐☐ *jin*

Section 9: Reproductive history (for women)

9.1·· How old were you when you had your first menstrual period?(if none put #, Go to **Q9.7**) ☐☐ 'ears

9.2 Have you had your menopause?

☐ No → ***If ticked,*** *Q9.2a Are you on your period today (☐Yes, ☐No), then* ***Go to Q9.3***

☐ Yes, currently

☐ Yes, had menopause → If so, **Q9.2b** age of completion of menopause: ☐☐ ɜar

9.2.1 Are you taking or have you taken any medications to relieve the symptoms associated with menopause? ☐ Yes, ☐ No; →If yes, **Q9.2.1a** please specify type of medication used:

Yes	No	
☐	☐	HRT
☐	☐	Traditional Chinese medicine
☐	☐	Other medication

9.3 How many times have you ever been pregnant? ☐☐ times

If none then Move to ***Q9.5***

9.3.1 Please give number for different pregnancy outcome:

a) Live birth: ☐☐ times → ***If none, Go to Q9.5***

b) Still birth: ☐☐ times

c) Spontaneous abortion: ☐☐ times

d) Induced abortion: ☐☐ times

9.4 Age and length of breastfeeding at each live birth (twins or more =one birth)?

Live Birth	**Q9.4a** Age at end of pregnancy	**Q9.4b** Months of breastfeeding	**Q9.4c** Twins or more Yes	No
1st	☐☐	☐☐	☐	☐
2nd	☐☐	☐☐	☐	☐
3rd	☐☐	☐☐	☐	☐
…	………	…...	…	…
Nth	☐☐	☐☐	☐	☐

9.5 Have you ever used oral contraceptive pills?

☐ Never → ***If ticked, Go to Q9.6***

☐ Past use → If ticked, **Q9.5a** age when you last stopped the pill: ☐☐ Year

☐ Current use

9.5.1 How old were you when you first used oral contraceptives? ☐☐ Year

9.5.2 For how long altogether have you used oral contraceptives? ☐☐ Year

9.6 Have you ever used a contraceptive coil?

- ☐ Never → ***If ticked, Go to Q9.7***
- ☐ Past use →if ticked, **Q9.6a** age when you last stopped using the coil? ☐☐ Year
- ☐ Current use

9.6.1 How old were you when you first used a coil? ☐☐ Year

9.6.2 For how long altogether have you used a coil? ☐☐ Year

9.7 Have you ever had any of the following procedures?

	Yes	No	If YES, age of operation?
a) Hysterectomy?	☐	☐	____________ Years
b) Removal of one or both ovaries?	☐	☐	____________ Years
c) Surgery to remove a breast lump?	☐	☐	____________ Years
d) Sterilization?	☐	☐	____________ Years
e) Caesarean Section?	☐	☐	____________ Years

Section 10: Sleeping, mood & mental situation

10.1 In general, how satisfied are you with your life?

☐ Very satisfied
☐ Satisfied
☐ Neither satisfied nor dissatisfied
☐ Unsatisfied
☐ Very unsatisfied

10.2 <u>See Appendix 4</u> for Attitude N scale B questionnaire)

10.2.1 Was the N Scale B questionnaire self-administered? ☐ Yes, ☐ No

---

10.3 Over the past two years have you had any of the following major events in your life?

Yes	No		Yes	No	
☐	☐	Marital separation/divorce	☐	☐	Major injury or traffic accident
☐	☐	Loss of job/retirement	☐	☐	Death / major illness of spouse
☐	☐	Business bankrupt	☐	☐	Death / major illness of other close family member
☐	☐	Violence	☐	☐	Major natural disaster (e.g. flood & drought)
☐	☐	Major conflict within family	☐	☐	Loss of income / living on debt

---

10.4 During the past month, did you have any of the following sleeping problem for ≥3 days each week?

Yes	No	
☐	☐	Taking >30 minutes to fall asleep after going to bed or waking up in the middle of the night
☐	☐	Waking up early and not being able to go back to sleep
☐	☐	Needing to take medicine (including herbal or sleeping pills) at least once a week to help sleep
☐	☐	The quality of sleep has adversely affect your daytime performance or activities

If ticked "No" to all the four questions, then *Go to <u>Q10.5</u>*

10.4.1 How long has your sleeping problem lasted? _______ Years

---

10.5 Do you usually take a daytime nap? ☐ Yes usually, ☐ Yes, only in certain season, ☐ No

10.6 Do you snore during sleep? ☐ Yes, Frequently, ☐ Yes, Sometimes, ☐ No / Don't know

---

10.7 Do you have to do nightshift regularly in your current or previous work?

☐ Yes, ☐ No; →If yes, **<u>Q10.7a</u>** how often: ☐ Daily, ☐ Weekly, ☐ Monthly; and **<u>Q10.7b</u>** for how many years _____ Years

---

10.8 How many hours do you typically sleep per day (incl. naps)? [ ][ ] Hours

---

10.9 During your entire life, have you had the following situations for 2 or more weeks continuously?

Yes	No	
☐	☐	Feeling much more sad, or depressed than usual
☐	☐	Loss of interest in most things like activities that usually give you pleasure
☐	☐	Being so hopeless that you had no appetite to eat even your favourite food
☐	☐	Feeling worthless and useless, everything went wrong was your fault and life is very difficult that there was no way out

(If answer "Yes" to any of the questions, then complete CIDI-A:Appendix 5, Section A)

10.10 During your entire life, have you experienced the following situations?

Yes	No	
☐	☐	Having a period lasting <u>one month or longer</u> when <u>most of time</u> you felt worried, tense, or anxious and it interfered your life ***(if ticked "Yes", then complete CIDI-B: Appendix 5, Section B)***
☐	☐	Having a pain or discomfort in your body lasting ≥3 months that interfered with your life
☐	☐	Having had a spell or an attack when all of sudden felt frightened, anxious, or very uneasy
☐	☐	Having had inexplicable strong fear in situations such as closed space (cave, elevator, airplane etc), in the crowds or public such that you would avoid such situations

---

Section 11: Physical examination

11.1 Standing height (without shoes) ……………………. [ ][ ][ ].[ ][ ] cm

11.2 Sitting height ………………………………………. [ ][ ][ ].[ ][ ] cm

11.3 Waist …………………………………………………… [ ][ ][ ].[ ][ ] cm

11.4 Hip ……………………………………………………… [ ][ ][ ].[ ][ ] cm

11.5 Hand grip strength ……………Right………………… [ ][ ].[ ][ ] kg

Left………………… [ ][ ].[ ][ ] kg

Staff code [ ][ ][ ]

11.6 Weight (without shoes, but in light clothing) ………. [ ][ ][ ].[ ] Kg

11.7 BMI……………………………………………………… [ ][ ].[ ][ ] kg/m^2

Staff code [ ][ ][ ]

For all these above questions (Q11.1~Q11.7), enter # if not measured.

11.8 Did you take any drugs to lower blood pressure in the last 2 days? ☐ Yes ☐ No

11.9 Do you have any of the peripheral artery disease symptoms including painful cramping legs, leg numbness or weakness, and cold legs? ☐ Yes ☐ No

11.10 Blood pressure & heart rate (to be measured after 5 minutes in the seated position)

	First	Second	
**SBP**	[ ][ ][ ]	[ ][ ][ ]	mmHg
**DBP**	[ ][ ][ ]	[ ][ ][ ]	mmHg
**Heart rate**	[ ][ ][ ]	[ ][ ][ ]	bpm

Ankle blood pressure (enter # if not measured)

	First	Second	
**SBP**	[ ][ ][ ]	[ ][ ][ ]	mmHg
**DBP**	[ ][ ][ ]	[ ][ ][ ]	mmHg

Staff code [ ][ ][ ]

11.11 Hours since last ate or drank anything (ignore non-caloric drinks such as water and tea)? _______ **hours**

Staff code [ ][ ][ ]

11.12 Blood sample collected: Yes ☐ Failed ☐

Staff code [ ][ ][ ]

11.13 Urine sample collected: Yes ☐ No ☐

11.14 Lung function & COex levels (enter # if not measured):

	First	Second	
**COex**……………………	☐☐	…..☐☐	ppm
**%COHB** …………………	☐☐.☐☐	..☐☐.☐☐	**%**
**FEV1**	☐.☐☐	☐.☐☐	Liter
**FVC**	☐.☐☐	☐.☐☐	Liter

Staff code ☐☐☐

11.15 Assessment of subject's cooperation and the reliability of data collected?

a) Assessment of subject's cooperation?

☐ Good

☐ Fair

☐ Poor

b) Assessment of the reliability of the information collected?

☐ Good

☐ Fair

☐ Poor

Date of interview _____Year______Month___Day, **Signature of interviewer**________

Appendix 1: List of province in mainland China (plus other and don't know)

1. Anhui
2. Beijing
3. Chongqing
4. Fujian
5. Gansu (local province for RC58, Tianshui, Gansu)
6. Guangdong
7. Guangxi (local province for RC46, Liuzhou)
8. Guizhou
9. Hainan (local province for RC26, Haikou)
10. Hebei
11. Heilongjiang (local province for RC16, Haerbin)
12. Henan (local province for RC68, Huixian, Henan)
13. Hubei
14. Hunan (local province for RC88, Liuyang, Hunan)
15. Jiangsu (local province for RC36, Suzhou)
16. Jiangxi
17. Jilin
18. Liaoning
19. Nei Mongol
20. Ningxia
21. Qinghai
22. Shaanxi
23. Shandong (local province for RC12, Qingdao)
24. Shanghai
25. Shanxi
26. Sichuan (local province for RC52, Sichuan)
27. Tianjin
28. Xinjiang
29. Xizang
30. Yunnan.
31. Zhejiang (local province for RC78, Tongxiang, Zhejiang)
32. Others (eg, Hong Kong, Macau, Taiwan)
33. Don't know

Note: unlike most questions, coding for this question is one-based, as shown, e.g. 1 = Anhui, 2 = Beijing etc.

Note: The order of provinces name in LTFollow up does not seem to have any logics (eg, PINYIN or Chinese words). So, for easiness of use I would prefer to use the present list. In addition, there are also two new area codes compared with that in LTFollow up (code 66 is not a province name and for the present questionnaire needs to be removed).

Appendix 2: Drug list

List 1: for diabetes

1. Aspirin
2. ACE-I
3. Beta-blocker
4. Statins
5. Diuretics
6. Ca^{++} antagonist
7. Clopidogrel
8. ARB
9. Other anti-hypertensive drugs
10. Traditional Chinese medicine
11. Chlorpropamide or metformin (oral tablets)
12. Insulin (injection)

List 2: for MI/angina/other IHD/stroke/hypertension

1. Aspirin
2. ACE-I
3. Beta-blocker
4. Statins
5. Diuretics
6. Ca^{++} antagonist
7. Clopidogrel
8. ARB
9. Other anti-hypertensive drugs
10. Traditional Chinese medicine

Appendix 3: COPD questionnaire (Q7.5)

The following questions are related to your chronic lung disease. Please give the answers you feel most appropriate.

1. **Overall, how would you rate the severity of your chronic lung disease now?**
   - ☐ Very severe
   - ☐ Severe
   - ☐ Moderate
   - ☐ Mild
   - ☐ Very mild

2. **How does your doctor classify the severity of your condition?**
   - ☐ Stage1 (mild)
   - ☐ Stage 2 (moderate)
   - ☐ Stage 3 (severe)
   - ☐ Stage 4 (very severe)
   - ☐ Doctor has not diagnosed or told me
   - ☐ Don’t know

3. **The next part of the questionnaire is to measure the impact of respiratory symptoms on wellbeing and your daily life. Please indicate on a scale of 0 to 5 how strongly you feel about the two pair of the statements related to each question.**

	Score					
	**0**	**1**	**2**	**3**	**4**	**5**
	☐	☐	☐	☐	☐	☐

1) **Frequency of your cough?**
   0= Never cough; 5= Cough all the time

2) **Amount of mucus in your chest?**
   0= No mucus at all; 5= Full of mucus

3) **Tightness of your chest?**
   0= Not tight at all; 5= Very tight

4) **Breathless when walking up a hill/one flight of stairs?**
   0= Not breathless; 5= Very breathless

5) **Limitation of daily activities at home by the condition?**
   0= Not limited; 5= Very limited

6) **Degree of confidence about leaving your home?**
   0= Confident; 5= Not confident at all

7) **Quality of sleep at night and whether it is affected by the condition?**
   0= I sleep soundly; 5= I don’t sleep soundly because of my lung condition

8) **Usual levels of energy?**
   0= I have lots of energy; 5= I have no energy at all

4. **Have you ever had your lung function tested by blowing hard into a tube (excluding the present and previous CKB tests)?**

- ☐ Yes
- ☐ No
- ☐ Don't know

5. **Have you ever had your lung function tested in a cabin, called a phlethysmograph?**

- ☐ Yes
- ☐ No
- ☐ Don't know

6. **In the past 12 months what types of health care professionals have you seen about your condition?**

Yes	No	
☐	☐	General physician
☐	☐	Respiratory specialist
☐	☐	Cardiologist/heart specialist
☐	☐	Traditional Chinese medicine doctor
☐	☐	Local health centre or village doctor
☐	☐	Other medical professional

7. **In the past 12 months, how many times have you been hospitalised overnight, or longer, as a direct result of your condition?** _____ times (If none, put 0)

8. **In the past 12 months, have you taken any of the following to treat worsening of your breathing problems?**

Yes	No		
☐	☐	Antibiotics	} → ***If ticked "NO" for all items, Go to <u>Q9.</u>***
☐	☐	Oral steroids	
☐	☐	Injectable steroids	

8.1 **In the past 12 months, how many episodes of COPD exacerbation have you had when you took antibiotics, oral steroids, or injectable steroids to treat worsening of your breathing problems? (if none, put 0)** _____ times

9. **In the past seven days, have you taken any prescriptive medicine (such as inhalers, tablets or something else) for the condition?**

- ☐ Yes → ***If ticked, Go to <u>Q9.1</u>***
- ☐ No
- ☐ Don't know

9.1 **What types of medication have you taken in the past seven days?**

Yes	No	
☐	☐	Short-acting beta-agonists (e.g. albuterol, salbutamol)
☐	☐	Short-acting anti-muscarinic agents (e.g. Atrovent, Combivent
☐	☐	Long-acting beta-agonists (salmeterol/Serevent, formoterol/Foradil)

☐ ☐ Long-acting anti-muscarinic agent (tiotropium/Spiriva)
☐ ☐ Oral bronchodilators
☐ ☐ Long-acting beta agonist+inhaled corticosteroid combination inhalers
☐ ☐ Inhaled corticosteroids
☐ ☐ Traditional Chinese medicine
☐ ☐ Other treatments

10. In the past 12 months have you used home oxygen for your condition?

☐ Yes → ***If ticked, Go to Q10.1***
☐ No
☐ Don't know

10.1 About how often do you use oxygen at home?

☐ Every day
☐ Most days
☐ Once a week
☐ Once a month
☐ Only occasionally

11. In the past 12 months, have you had an influenza vaccine (also called a flu shot)?

☐ Yes
☐ No
☐ Don't know

12. In the past 12 months, have you had a vaccine or shot which protects you against pneumonia?

☐ Yes
☐ No
☐ Don't know

13. How well informed do you feel you are about your condition and its treatment?

☐ Well informed
☐ Less than adequately informed
☐ Very poorly informed
☐ Don't know

Appendix 4: N scale B

I am going to give you a set of questions about the way of your thinking and doing things. These questions have no right or wrong answers. Please answer each question by choosing either YES or NO. The best answer is usually the one that comes to mind first, so work quickly and do not think too long about each question. If you are having a hard time making up your mind about a question, ask yourself: Is this more true for me (if so, answer YES) or more false for me (if so, answer NO).

Q1 Do you often worry about things you should not have done or said?	1. Yes □ 0. No □
Q2 Are you an irritable person?	1. Yes □ 0. No □
Q3 Are your feelings easily hurt?	1. Yes □ 0. No □
Q4 Do you often feel "fed-up"?	1. Yes □ 0. No □
Q5 Are you often troubled about feelings of guilt?	1. Yes □ 0. No □
Q6 Does your mood often go up and down?	1. Yes □ 0. No □
Q7 Would you call yourself a nervous person?	1. Yes □ 0. No □
Q8 Are you a worrier?	1. Yes □ 0. No □
Q9 Do you worry about awful things that might happen?	1. Yes □ 0. No □
Q10 Would you call yourself tense or highly-strung?	1. Yes □ 0. No □
Q11 Do you worry about your health?	1. Yes □ 0. No □
Q12 Do you suffer from sleeplessness?	1. Yes □ 0. No □
Q13 Have you often felt listless and tired for no reason?	1. Yes □ 0. No □
Q14 Do you often feel life is very dull?	1. Yes □ 0. No □
Q15 Do you worry a lot about your looks?	1. Yes □ 0. No □
Q16 Have you ever wished that you were dead?	1. Yes □ 0. No □
Q17 Do you ever feel "just miserable" for no reason?	1. Yes □ 0. No □
Q18 Do you worry too long after an embarrassing experience?	1. Yes □ 0. No □
Q19 Do you suffer from “nerves”?	1. Yes □ 0. No □
Q20 Do you often feel lonely?	1. Yes □ 0. No □
Q21 Are you easily hurt when people find fault with you or the work you do?	1. Yes □ 0. No □
Q22 Are you sometimes bubbling over with energy and sometimes very sluggish?	1. Yes □ 0. No □
Q23 Are you touchy about some things?	1. Yes □ 0. No □

Appendix 5: CIDI-SF A & B

SECTION A: MAJOR DEPRESSIVE EPISODE

The next set of questions cover experiences you may have had at any point in your life. Since these questions cover a long time period, please take your time to think over your entire life before answering*.*

A 1. During your entire life, was there ever a time when you felt sad, blue, or depressed for two weeks or more in a row?

1. YES

0.NO

A1.1. During your entire life, was there ever a time lasted 2 weeks or more when you had a negative feeling about yourself such as everything went wrong was you fault, you were useless, life was so difficult for you that there was no way out？

1. YES

0. NO

A1.2. During your entire life, was there ever a time lasted 2 weeks or more when you felt so exhausted or even numb

1.YES

0.NO

A1.3 During your entire life, was there ever a time lasting two weeks or more you lost interests in most hobbies, work or activities you used to enjoy such as TV, music, sport, holiday, eating out, or even you had to participated in these events you showed no interests in them?

1. YES

0. NO

THANK YOU! (The end)

A1a. For the next few questions, please think of <u>the two-week period</u> during your entire life when these feelings were <u>worst</u> (if A1=1 or A1.1=1 or A1.2=1)/ when you had the most complete loss of interest in things (if A1.3=1). During that time did the feelings of being sad, blue, or depressed (if A1=1 or A1.1=1 or A1.2=1)/did the loss of interest (if A1.3=1) usually last <u>all day long</u>, <u>most</u> of the day, <u>about half</u> the day, or <u>less than half</u> the day?

0. ALL DAY LONG → (continue)
1. MOST → (continue)
2. ABOUT HALF → THANK YOU! (The end)
3. LESS THAN HALF → THANK YOU! (The end)

A1b. During those two weeks, did you feel this way <u>every day</u>, <u>almost every day</u>, or <u>less often</u>?

0. EVERY DAY → (continue)
1. ALMOST EVERY DAY → (continue)
2. LESS OFTEN → THANK YOU! (The end)

Page break (optional)

A1c. <u>During those two weeks</u> did you lost interests in most hobbies, work or activities you used to enjoy such as TV, music, sport, holiday, eating out, or even you had to participated in these events you showed no interests in them?

1. YES
0. NO

A1d. Thinking about those same two weeks, did you feel more tired out or low on energy than is usual for you?

1. YES
0. NO

Page Break (optional)

A2. Did you gain or lose weight without trying, or did you <u>stay about the same</u>?

Interviewer: If R asks: "Are we still talking about the same two weeks?" Answer: "Yes."

0. GAIN → A2a
1. LOSE → A2a
2. IF VOLUNTEERED: BOTH GAINED AND LOST WEIGHT → A2a
3. STAY ABOUT THE SAME → (continue)
4. IF VOLUNTEERED R WAS ON A DIET → (continue)

A2a. About how much did you gain/you lose/ your weight change?

_________ JIN (accept range of value, e.g, 5-8, using the average)
<u>Minimum: >=1</u>
<u>Maximum: warning if entry >=100 JIN</u>
<u>Data type: Integer or 1 decimal place (only allow 0.5 for half Jing)</u>

A3. Did you have more trouble falling asleep than you usually do during those two weeks?

1. YES | 0. NO → GO TO A4

A3a. Did that happen <u>every night</u>, <u>nearly every night</u>, or <u>less often</u> during those two weeks?

0. EVERY NIGHT | 1. NEARLY EVERY NIGHT | 2. LESS OFTEN

A4. During those two weeks, did you have a lot more trouble concentrating than usual?

Interviewer: If R asks: "Are we still talking about the same two weeks?" Answer: "Yes."

1. YES | 0. NO

A5. People sometimes feel down on themselves, no good, or worthless. During that two week period, did you feel this way?

1. YES | 0. NO

Interviewer: If R asks: "Are we still talking about the same two weeks?" Answer: "Yes."

A5.1 During those two weeks, did you feel hopeless about things?

1. YES | 0. NO

Interviewer: If R asks: "Are we still talking about the same two weeks?" Answer: "Yes."

A6. Did you think a lot about death -- either your own, someone else's, or death in general during those two weeks?

Interviewer: If R asks: "Are we still talking about the same two weeks?" Answer: "Yes."

1. YES | 0. NO

A7a. Did you have a plan to harm yourself on purpose during those two weeks?

1. YES | 0. NO

A7b. Did you take any action to harm yourself on purpose during those two weeks?

1. YES | 0. NO

CHECKPOINT -- (COUNT YES RESPONSES IN A1-A7)

1. ZERO QUALIFYING RESPONSES GOTO End (Thank you!)

Qualifying Responses: A1c=1, A1d=1, A2a >=10Jin, A3a=0 or 1, A4=1, A5=1, A5.1=1; A6=1; A7a=1; A7b=1

2. IF ONE OR MORE QUALIFYING RESPONSE GO TO A8

A8. To review, you had two weeks in a row during your entire life,
(IF “YES” to A1) then “when you were sad, blue, or depressed, also you had some other feelings or problems like”… (READ UP TO FIRST 3 QUALIFYING RESPONSES)

(IF “YES” TO A1.1) then “when you had a negative feeling about yourself such as everything went wrong was you fault, you were useless, life was so difficult for you that there was no way out and also had some other feelings or problems like”… (READ UP TO FIRST 3 QUALIFYING RESPONSES)

(IF “YES” to A1.2) then “when you felt so exhausted or even numbers that you would burst into tears without a reason, also you had some other feelings or problems like”… (READ UP TO FIRST 3 QUALIFYING RESPONSES)

(IF “YES” to A1.3) then “when you lost interest in most things like hobbies, work, or activities that usually give you pleasure, also you had some other feelings or problems like”… (READ UP TO FIRST 3 QUALIFYING RESPONSES)

About how many years altogether did you feel this way during your entire life?

________________# OF YEARS → ENTER WKS. IF <1 YEAR

Minimum value: 2 WKS (if<1 year) OR 1 YEAR; Maximum value: 51 WEEKS (if <1 years) OR current age; Data type: integer (other values unacceptable)

A8.1 How old were you when you had your first episode of depression -- that is, when you felt sad or not interested in things for at least two weeks and had several of the other symptoms you described above? Age:______

Minimum value: 0; Maximum value: current age

A9. Think about this most recent time when you had two weeks in a row when you felt this way. How long ago was that?

____________________MONTHS in the past (if at present, enter “0”; If less than 1 month, round up to half month and enter 0.5)

Minimum value: 0; Maximum value: 12 x age; Data type: integer or 1 decimal place (only allow 0.5for half month)

A9.1. How many times you feel like this, that is, when you felt sad or not interested in things for at least two weeks, in your life? ____

Minimum value: 0; Maximum value: 999

Page Break (optional)

A10. Did you tell a doctor about these problems? (By "doctor" I mean either a medical doctor or osteopath, or a student in training to be either a medical doctor or osteopath, i.e., medical staff working in a hospital.)

1. YES 0. NO

A11. Did you tell any other professional (such as a psychologist, social worker, counsellor, nurse, Clergy, or other helping professional working in non-hospital environment)?

1. YES 0. NO

A12. Did you tell your family members or close friends or relatives?

1. YES 0. NO

A13. Did you take medication or use drugs or alcohol more than once for these problems?

1. YES 0. NO

A14. Did you take any treatments for your condition? (More than one answer can be selected)

Treatments	YES (1)	NO (0)
Psychiatric		
Herbal medicine		
Vitamin or other health products		

A15. How much did these problems interfere with your life or activities -- a lot, some, a little, or not at all

0. A LOT	1. SOME	2. A LITTLE	3. NOT AT ALL

THANK YOU!
(The end)

SECTION B: GENERALIZED ANXIETY DISORDER

The next set of questions cover experiences you may have had at any point in your life. Since these questions cover a long time period, please take your time to think over your entire life before answering.

B1. During your entire life, did you ever have a period lasting one month or longer when most of the time you felt worried, tense, or anxious?

1. YES

0. NO

B1a. People differ a lot in how much they worry about things. Did you have a time in your entire life when you worried a lot more than most people would in your situation?

1. YES

0. NO → THANK YOU! THE END

B2. Has that period ended or is it still going on?

0. ENDED → B2a. How long did it go on before it ended?

1. STILL GOING ON → B2b. How long has it been going on?

0. Less than 1 year → How many months;_____

1. More than 1 year → How many years___

2. All my life or as long as I can remember

Minimum value: 1;
Maximum value: 12;
Data type: integer
Decimal place: 1 (only allow 0.5 for half month)

Minimum value: 1;
Maximum value: 99, warning if entry >=50 year
Data type: integer
Decimal place: 1 (only allow 0.5 for half year)

INTERVIEWER CHECKPOINT

1. B2a/B2b IS SIX MONTHS OR LONGER, OR R VOLUNTEERED: "ALL MY LIFE" OR "AS LONG AS I CAN REMEMBER"

THANK YOU (THE END)

B3. (During that period, was your/is your) worry stronger than in other people?

1. YES   0. NO

B4. (Did/Do) you worry most days?

1. YES   0. NO

B5. (Did/Do) you usually worry about one particular thing, such as your job security or the failing health of a loved one, or more than one thing?

0. ONE THING   1. MORE THAN ONE THING

B6. (Did/Do) you find it difficult to stop worrying?

1. YES   0. NO

B7. (Did/Do) you ever have different worries on your mind at the same time?

1. YES   0. NO

B8. How often (was/is) your worry so strong that you (couldn't/can't) put it out of your mind no matter how hard you (tried/try) -- often, sometimes, rarely, or never?

0. OFTEN   1. SOMETIMES   2. RARELY   3. NEVER

B9. How often (did/do) you find it difficult to control your worry -- often, sometimes, rarely, or never?

0. OFTEN   1. SOMETIMES   2. RARELY   3. NEVER

B10. What sort of things (did/do) you mainly worry about?

	YES (1)	NO (0)
1. Relationship (spouse, partner, ex-spouse)?		
2. Other family members (including members of spouse family)?		
3. Own children (including step-children, grown up children) or parents (including step-parents)?		
4. Job (course study)?		

	YES (1)	NO (0)
5. Social and/or interpersonal relationships?		
6. Housing/economic situation		
7. Problems other than specified above?		
8. Some terrible things will happen (but nothing has happened yet)		

B11. When you (are/were) worried or anxious, (are/were) you also

	YES (1)	NO (0)
1. Restless?		
2. keyed up or on edge?		
3. easily tired?		
4. have difficulty keeping your mind on what you were doing?		
5. more irritable than usual?		
6. have tense, sore or aching muscles		
7. have trouble falling asleep or staying asleep?		

CHECKPOINT

1. 0-1YES RESPONSES IN THE B11 SERIES; 2. ALL OTHERS

THANK YOU (The End)

B12. Did you tell a doctor in hospital about these problems? (By "doctor" I mean either a medical doctor or osteopath, or a student in training to be either a medical doctor or osteopath, i.e., medical staff working in a hospital.)

1. YES 0. NO

B13. Did you tell any other professional (such as a psychologist, social worker, counsellor, nurse, Clergy, or other helping professional working in non-hospital environment)?

1. YES 0. NO

B14. Did you tell your family members or close friends or relatives about these problems?

1. YES 0. NO

B15. Did you take medication or use drugs or alcohol more than once for these problems?

1. YES 0. NO

B16. Did you take any treatments for your condition?

Treatments	YES (1)	NO (0)
Psychiatric		
Herbal medicine		
Vitamin or other health products		

B17. How much did these problems interfere with your life or activities -- <u>a lot</u>, <u>some</u>, <u>a little</u>, or <u>not at all</u>

0. A LOT 1. SOME 2. A LITTLE 3. NOT AT ALL

THANK YOU!
(The end)
