# Supplementary material for: Metabolically healthy obesity, transition to unhealthy metabolic status, and vascular disease in Chinese adults: A cohort study
Source: PLoS Med. 2020 Oct 30;17(10):e1003351. doi: 10.1371/journal.pmed.1003351 (PMC7598496; doi:10.1371/journal.pmed.1003351)
Supplement: Attachment — Submitted filename: Response letter.docx [file pmed.1003351.s013.docx]

Dear Editor,

Manuscript PMEDICINE-D-20-00639R1

**Metabolically healthy obesity, transition to unhealthy metabolic status and vascular disease in 0.5 million Chinese adults: a prospective cohort study**

Thank you for your comments on the above manuscript and for the opportunity to revise it. We have taken the reviewers’ comments and suggestions into careful consideration and revised the manuscript accordingly. On the following pages, please find our point-to-point responses to the reviewers’ concerns in the order that they were originally listed, and details of the pages on which the changes have been made.

Sincerely,

Prof. Tao Huang,

Department of Epidemiology & Biostatistics,

School of Public Health, Peking University, China.

38 Xueyuan Road, Bejing, 100191 China

Phone: (86) 010-82801528

Email: huangtaotao@pku.edu.cn

**Response to Reviewers’ comments**

We have revised the manuscript in light of helpful comments from editors and three reviewers. Our responses to the specific comments raised are provided below, along with the line numbers of the related text in the revised manuscript.

**Editors**

1. Title: Please revise your title according to PLOS Medicine's style.

**Response:** Thank you. We have revised the title to “Metabolically healthy obesity, transition to unhealthy metabolic status and vascular disease in 0.5 million Chinese adults: a prospective cohort study”.

2. Data – you say some restrictions will apply, then say all data is available in the MS and Supp Files. Please state what data is available and what is not and why.

**Response:** We have revised it in data availability. As you can see in data sharing, “details of how to access China Kadoorie Biobank data and details of the data release schedule are available from [www.ckbiobank.org/site/Data+Access](http://www.ckbiobank.org/site/Data+Access)”. (line 517-519, page 24)

3. Abstract – this should be formatted with 3 sections: Background, Method and Findings, Conclusion. Please format accordingly.

**Response:** We have revised it accordingly.

4. Abstract – use months as well as years for recruitment and tell us the cities / regions where recruitment took place, if known; Please use 95% Cis as well as p values for all quantifiable data (both here and throughout the manuscript); Please ensure summary demographic information is provided, including gender ratio and mean age and number of smokers, etc; Please provide – as the last sentence of the ‘Methods and Findings’ section a sentence on the limitations of the study;

**Response:** We have revised the recruitment time and places (line 38-40, page 2). We have added p values as well as 95% CIs. The summary demographic information is provided (line 47-49, page 2). Furthermore, we have added the limitations of the study (line 57-59, page 2).

5. At this stage, we ask that you include a short, non-technical Author Summary of your research to make findings accessible to a wide audience that includes both scientists and non-scientists. The Author Summary should immediately follow the Abstract in your revised manuscript. This text is subject to editorial change and should be distinct from the scientific abstract. Please see our author guidelines for more information: https://journals.plos.org/plosmedicine/s/revising-your-manuscript#loc-author-summary

**Response:** We have added the author summary. (line 66-91, page 3-4)

6. Refs in main text, these need to be presented in square brackets and not using superscript. In the main text where you use 95%Cis, please also add p values. Please include line numbers on your revised version.

**Response:** We have presented references in main text in square brackets and not using superscript. We have also added p values and line numbers.

7. Please be careful of deriving cause where non can be drawn as this is not a trial: “Our data provide evidence…” and “In summary, our study provides evidence that obesity, even without metabolic syndrome…”

**Response:** We have changed “provides evidence” to “shows”. (line 330, page 15 and line 441, page 20)

8. Please use the "Vancouver" style for reference formatting, and see our website for other reference guidelines https://journals.plos.org/plosmedicine/s/submission-guidelines#loc-references (noting remove ital font specifically)

**Response:** We have revised it accordingly.

9. Did your study have a prospective protocol or analysis plan? Please state this (either way) early in the Methods section.

a) If a prospective analysis plan (from your funding proposal, IRB or other ethics committee submission, study protocol, or other planning document written before analyzing the data) was used in designing the study, please include the relevant prospectively written document with your revised manuscript as a Supporting Information file to be published alongside your study, and cite it in the Methods section. A legend for this file should be included at the end of your manuscript.

b) If no such document exists, please make sure that the Methods section transparently describes when analyses were planned, and when/why any data-driven changes to analyses took place.

c) In either case, changes in the analysis-- including those made in response to peer review comments-- should be identified as such in the Methods section of the paper, with rationale.

**Response:** We have added the study analysis plan (S1 Analysis plan).

10. Please ensure that the study is reported according to the STROBE guideline, and include the completed STROBE checklist as Supporting Information. Please add the following statement, or similar, to the Methods: "This study is reported as per the Strengthening the Reporting of Observational Studies in Epidemiology (STROBE) guideline (S1 Checklist)."

The STROBE guideline can be found here: http://www.equator-network.org/reporting-guidelines/strobe/

When completing the checklist, please use section and paragraph numbers, rather than page numbers.

**Response:** We have added STROBE guideline (S1 Checklist).

**Reviewer 1**

**Comments to the Author**

The main issue is that the authors categorize all the independent variables. This is a mistake. In *Regression Modeling Strategies* Frank Harrell lists 11 problems with this and sums up "Nothing could be more disastrous". I wrote a blog post that shows some of the problems, graphically: https://medium.com/@peterflom/what-happens-when-we-categorize-an-independent-variable-in-regression-77d4c5862b6c

Instead, BMI, waist circumference and so on should be left continuous and splines used to look for nonlinearities. In addition, metabolic health status should be a continuous variable based on a combination of the factors listed on p. 6. One way to get such a measure would be factor analysis. (Categories here *might* be useful for transitions, although I think continuous is better there, too; but for the regressions, continuous IVs are definitely better).

**Response:** Thank you for your thoughtful comments and suggestions. We have added sensitivity analyses as suggested in supplemental tables (S4 Fig., S5 Fig.). “We found systolic blood pressure, diastolic blood pressure, and random plasma glucose had non-linear association with major vascular disease (p<0.05) and no evidence of non-linear association between waist circumference and major vascular disease (p=0.088) (S4 Fig). We found higher risk of major vascular disease with the increase of number of metabolic disorders (S5 Fig.).”(line 285-289, page 13) However, in previous report published in Lancet Diabetes Endocrinol [1] and Circulation[2] journals, metabolic health status were also defined by presence of metabolic disorders or metabolic syndrome.

Also, BMI is a poor measure of adiposity, even if you don't categorize. Waist hip ratio is much better. BMI has the advantage of being easier to measure, but since you already measured waist, why did you ignore hips? Waist hip ratio is a good measure of adiposity.

**Response:** Thank you for your great comments. In this study, BMI is thought to be an indicator of general obesity, while we agree that waist circumference and waist hip ratio are more related with body fat distribution and metabolic health status. Thus, we defined metabolic health based on a common definition of the metabolic syndrome, with elevated waist circumference one of the criteria[3]. As the reviewer suggested, we have added sensitivity analysis using waist hip ratio instead of waist circumference in supplemental table 1. The sensitivity analysis showed similar results. (line 250-254, line 304-308)

**Major comment:**

1. p. 5 Why only measure waist size? Why not adjust for height? (I know there are some studies using these cutoffs, but surely a 90 cm waist on a 2 meter tall man is not the same as one on a 1.7 m tall man.

**Response:** We have added sensitivity analysis using waist height ratio instead of waist circumference in supplemental table 1. (line 250-254, line 304-308)

2. Why were these glucose levels used?

**Response:** We defined metabolic health based on a common definition of the metabolic syndrome, with elevated plasma glucose one of the criteria[3]. In this study, random plasma glucose was collected and the time passed since participants last ate was recorded. Besides, participants with a glucose level ≥7.8 mmol/L and <11.1 mmol/L were invited to return the following day for fasting plasma glucose testing. Thus, we defined elevated plasma glucose as “fasting plasma glucose (FPG) ≥5.6 mmol/L or random plasma glucose (RPG) ≥11.1 mmol/L or self-reported diabetes”[3, 4].

3. p. 7 Near the bottom, what does "if appropriate" mean?

**Response:** As you can see in the note of table 1, “Baseline and the second re-survey characteristics of the study population were described adjusted for age, sex and region except for number of participants, age, sex and urban region”. “If appropriate” means except for age, sex, and urban region. For age and sex, they were not adjusted. For urban region, it was adjusted for age and sex. To avoid misunderstanding, I have added the explanation in the main text. (line 219, page 10)

4. p. 8 As noted, none of the continuous IVs should be categorized (food intake, income, exercise)

**Response:** Thank you for your thoughtful comments and suggestions. We have added sensitivity analysis controlling food intake and exercise in models as continuous variable in supplemental tables (S1 Table, S2 Table). However, in the questionnaire, income was asked as categorical variables. The sensitivity analysis showed similar results. (line 250-254, line 304-308)

5. Figure 1 might be better as a mosaic plot

**Response:** We have changed Figure 1 to a mosaic plot and added the description “The prevalence of MHO was higher in women than in men and in young people than in old people at baseline and the second re-survey” (line 268-270, page 13).

6. Figure 4 for this, categories might be useful. If you use categories, figure 4 should be a table with initial status and final status crossed and frequencies in the cells. A visual representation could be a mosaic plot. If you use continuous measures, you could make a scatterplot with a loess or other smoother.

**Response:** Thank you for your thoughtful suggestions. We have presented the changes from baseline to the second re-survey in Table 3.

**Reviewer 2**

**Major comment:**

1.	The study is largely confirmatory, even if larger-scale cohort studies are so far lacking from China. That MHO defined by the absence of the metabolic syndrome is still related to an increased risk have been shown by meta-analyses of several cohort studies. Also, it is well described that an unhealthy phenotype strongly increases risk across all BMI categories. Furthermore, that changes to an unhealthy phenotype increases risk has also been described before. Thus, the study lack novelty overall.

**Response:** Thank you for your comments. We acknowledge that some studies reported MHO individuals had higher risk of CVD. However, the prevalence of MHO showed large variation (MHO prevalence seemed to be higher in population from Asia than Caucasian or multi-ethnic origin) [5]. In addition, there is limited prospective evidence on the association of obesity and metabolic health status and transition with incident cardiovascular disease in Asian people, especially Chinese. The novelty of the present study is to explore the transition of obesity and metabolic health and their relations with incident major vascular events, especially subtypes such as stroke, major coronary events, and ischemic heart disease.

2.	While the cohort is indeed large, the resurveys to update BMI and MH status only included a minor fraction of the original cohort. As a result, the analysis considering MH status at the 2nd resurvey as baseline is severely underpowered. With the very short follow-up (2-3 years) and low number of cases this analysis isn't informative (easily observable by the large CIs for HRs)

**Response:** Thank you for your great comments. We acknowledge that this is the limitation of our study, we have discussed this issue on page 20 (line 429-432). However, the analysis using data from the second re-survey was also done to validate the results of baseline. As we can see in figure 2, participants with MUO had a higher risk of developing MVE in the second re-survey, which is similar with the result in baseline.

3.	This problem also affects the analysis on MH changes. First, overweight and obese individuals were combined in this analysis, which limits interpretation. Furthermore, while transition to MU in overweight/obese can be evaluated, such a transition in normal-weight could not. It also remains unclear how a stable MHO status is related to risk. The HR for MHOO (1.10) is not significant, however, the effect size isn't materially different from the analysis using baseline MHO (1.08). Still, the lack of precision doesn't allow to make conclusions.

**Response:** We combined overweight and obese individuals for small sample size. (line 191-192) We acknowledge that the HR for stable MHOO is not precise to make the null conclusion, and we have added the limitation in the discussion (line 429-434, page 20). However, in this analysis, it’s clear that change from MHOO to MUOO and stable MUOO had considerable higher risk of CVD.

4.	From the study methods it remains unclear how complete assessment of case status in the cohort is. How complete is the population coverage of disease registries? What is the proportion of participants with active follow-up and how were self-reports verified?

**Response:** We added the percentage of participants loss to follow-up, coverage of disease registries and linked to health insurance database, and the results of adjudication of CVD cases. (line 197-209, page 9-10)

5.	The discussion on p. 22 highlights that it is a novel or different finding that individuals with MU have higher risk across all BMI groups. This is a false statement as many studies have found a similar picture before, as has been summarized in meta-analysis (Ref. 16) or described from large scale cohorts (e.g. Lasalle 2018 - one of the largest studies, other Refs e.g. 8)

**Response:** We acknowledge that previous studies have observed similar results for CVD. However, limited data has been reported for subtypes of CVD such as MVE and MCE. Importantly, some studies suggested metabolic status was not more valuable than BMI in identifying individuals at risk for CVD[4, 6, 7], others found higher risk of CVD in participants without metabolic health across BMI categories[1, 2, 8]. Our results among Chinese adults supported the latter.

6.	The authors use one definition, the metabolic syndrome, although this definition has been discussed to be not strict enough to detect a true low-risk group (risk factors can still be present). Particularly the large sample from the baseline cohort could be used to evaluate alternative definitions of MH, e.g. the absence of any metabolic syndrome component.

**Response:** We thank the reviewer for the thoughtful suggestion and instruction. Unfortunately, the participants with obesity and absence of any metabolic syndrome component are few (n=311, 0.06%), and the incident CVD cases is not enough for the analysis.

**Reviewer 3**

**Major comment:**

Abstract:

1. I am unclear after reading the methods what the BMI categories were, how MHO and MHN was defined, how changes in metabolic health status were defined, and what statistical analysis was done to examine risk

**Response:** We have added the definitions in the abstract. (line 41-47, page 2) As for statistical analysis in this study, cox proportional hazard models were used with age as the time scale and participants with metabolically healthy normal weight (MHN) as reference group, to estimate the hazard ratios (HRs) for incident CVD types by BMI-metabolic health status at baseline and the second re-survey. The corresponding 95% confidence intervals (CIs) of HRs were calculated by use of the floating absolute risk method to enable comparisons between any two categories. In addition, HRs and corresponding 95% CIs were also calculated for incident CVD types by changes of BMI-metabolic health status with the same model, and the the reference group is participants with stable MHN.

Introduction:

1. remove phrasing that labels people as "obese" rather should state "people with obesity"

**Response:** We have revised it.

2. MHO is itself a misnomer implying that obesity can be healthy. If this is warranted, the introduction could state this or can be in the discussion

**Response:** We have added it in the introduction (line 99-100, page 5), and we have stated that “MHO was not a harmless condition” in the discussion (line 369, page 17).

3. Instead of body shape, would consider rephrasing to say "adiposity distribution"

**Response:** Thank you for your suggestion. We have changed “body shape” to “adiposity distribution”. (line 108, page 5)

4. Both in the abstract and in the introduction, the following words are used interchangeably: CVD, vascular risk, MVE, MCE, cardiovascular events. I think you should pick one and be consistent throughout unless they represent different things that are not well defined and are confusing. The most traditional one to use would be to consistently refer to all events as cardiovascular disease (CVD) and refer to each subtype as indicated.

**Response:** CVD, MVE, and MCE are different in ICD-10 codes. In this study, we focused the risk of MVE, MCE, IHD, and stroke, which are types of CVD. We have clarified this issue in Introduction section on page 6, line 119-121.

5. It would be nice to reference the recent BRICS study in Circulation about changing rates of CVD mortality in China and other low and middle-income countries and how obesity/diabetes may play a role in the first paragraph.

**Response:** Thank you for your suggestion. We have added the reference “Cardiovascular disease (CVD) is a leading cause of death and disability worldwide and contributes to more than 17 million deaths annually, especially 8.4 million CVD deaths across the BRICS (Brazil, Russia, India, China, and South Africa) in 2016.” (line 94-95, page 5).

Methods:

1. For the reader, please define and provide a reference as to why you chose the BMI cutoffs that you did. these may be appropriate for individuals of Asian ancestry, but they are not universal.

**Response:** Thank you for your thoughtful suggestion. We have added the reference (line 167, page 8).

Discussion:

1. Instead of 0.5 million just state the sample size or approximately 500,000

**Response:** We have substituted “0.5 million” with “approximately 500,000”. (line 349, page 16)

2. Could be shortened, specifically the paragraph on page 12 that extends to page 13 is too long.

**Response:** We have shortened the paragraph on page 16-17.

3. Paragraph that begins on page 14 should be rephrased to say "our findings show.." not the past tense

**Response:** We have revised it. (line 363, page 17)

4. The claim on page 14, that no other Asian cohort study has investigated the transition of individuals with MHO is not true. in the Multi-Ethnic Study of Atherosclerosis, patients of Chinese ancestry were included and they have studied this. Would revise and include this. However, the current study has a much larger sample size.

**Response:** We have revised it. (line 382, page 18)

5. The paragraph on page 15 is also far too long and could be cut in half.

**Response:** We have cut the paragraph in half on page 18-19.

6. Limitations should include the fact that no direct measure of visceral adiposity of %body fat was included and the discussion around how we know that BMI is a poor measure of obesity

**Response:** We have added the limitation (line 434-435, page 20).

7. The limitation of salt intake seems a bit odd since we are not talking about blood pressure anywhere. I would include the limitation of not having measured HbA1c which is the clinically relevant way to measure presence of pre-diabetes and diabetes

**Response:** In this cohort study, the exposure “metabolic health status” included the criterion of hyperglycemia, thus we didn’t consider presence of pre-diabetes and diabetes as a covariate. We have changed “salt intake” to “anti-hypertension drugs use and level of HbA1c” (line 437-438, page 20).

**Reference**

1.	Eckel N, Li Y, Kuxhaus O, Stefan N, Hu FB, Schulze MB. Transition from metabolic healthy to unhealthy phenotypes and association with cardiovascular disease risk across BMI categories in 90 257 women (the Nurses' Health Study): 30 year follow-up from a prospective cohort study. The lancet Diabetes & endocrinology. 2018;6(9):714-24.

2.	Arnlov J, Ingelsson E, Sundstrom J, Lind L. Impact of body mass index and the metabolic syndrome on the risk of cardiovascular disease and death in middle-aged men. Circulation. 2010;121(2):230-6.

3.	Alberti KG, Eckel RH, Grundy SM, Zimmet PZ, Cleeman JI, Donato KA, et al. Harmonizing the metabolic syndrome: a joint interim statement of the International Diabetes Federation Task Force on Epidemiology and Prevention; National Heart, Lung, and Blood Institute; American Heart Association; World Heart Federation; International Atherosclerosis Society; and International Association for the Study of Obesity. Circulation. 2009;120(16):1640-5.

4.	Thomsen M, Nordestgaard BG. Myocardial infarction and ischemic heart disease in overweight and obesity with and without metabolic syndrome. JAMA internal medicine. 2014;174(1):15-22.

5.	Rey-Lopez JP, de Rezende LF, Pastor-Valero M, Tess BH. The prevalence of metabolically healthy obesity: a systematic review and critical evaluation of the definitions used. Obesity reviews : an official journal of the International Association for the Study of Obesity. 2014;15(10):781-90.

6.	Hinnouho GM, Czernichow S, Dugravot A, Nabi H, Brunner EJ, Kivimaki M, et al. Metabolically healthy obesity and the risk of cardiovascular disease and type 2 diabetes: the Whitehall II cohort study. European heart journal. 2015;36(9):551-9.

7.	Li L, Chen K, Wang AP, Gao JQ, Zhao K, Wang HB, et al. Cardiovascular disease outcomes in metabolically healthy obesity in communities of Beijing cohort study. International journal of clinical practice. 2018:e13279.

8.	Hosseinpanah F, Barzin M, Sheikholeslami F, Azizi F. Effect of different obesity phenotypes on cardiovascular events in Tehran Lipid and Glucose Study (TLGS). The American journal of cardiology. 2011;107(3):412-6.
