# Supplementary material for: Metabolically healthy obesity, transition to unhealthy metabolic status, and vascular disease in Chinese adults: A cohort study
Source: PLoS Med. 2020 Oct 30;17(10):e1003351. doi: 10.1371/journal.pmed.1003351 (PMC7598496; doi:10.1371/journal.pmed.1003351)
Supplement: Attachment — Submitted filename: Response letter.docx [file pmed.1003351.s014.docx]

Dear Editor,

Manuscript PMEDICINE-D-20-00639R2

**Metabolically healthy obesity, transition to unhealthy metabolic status and vascular disease in Chinese adults: a prospective cohort study**

Thank you for your comments on the above manuscript and for the opportunity to revise it. We have taken the reviewers’ comments and suggestions into careful consideration and revised the manuscript accordingly. On the following pages, please find our point-to-point responses to the reviewers’ concerns in the order that they were originally listed, and details of the pages on which the changes have been made.

Sincerely,

Prof. Tao Huang,

Department of Epidemiology & Biostatistics,

School of Public Health, Peking University, China.

38 Xueyuan Road, Bejing, 100191 China

Phone: (86) 010-82801528

Email: huangtaotao@pku.edu.cn

**Response to Reviewers’ comments**

We have revised the manuscript in light of helpful comments from editors and three reviewers. Our responses to the specific comments raised are provided below, along with the line numbers of the related text in the revised manuscript.

**Academic Editor**

**Comments to the Author**

This study is very interesting, investigating the association of transition state from healthy overweight or obesity to unhealthy overweight or obesity with CVDs. If an overweight or obese participant had no metabolic disorders, the risks of CVDs would not increase. But, if an overweight or obese participant accompanied with metabolic disorders, the risks of CVDs would increase. The results suggested overweight or obese persons should not only pay attention to their weight or BMI, but also need to check the metabolic indexes regularly.

**Response:** Thank you for your positive comments. The point-to-point responses to the questions or suggestions are provided.

**Major comment:**

1. The sample was around 0.5 million for the baseline. However, the sample of resurvey was only close to 20 thousand. It was said that this represented a 5% random sampling from the baseline sample. So, please clarify the representativeness of the resurvey sample with the baseline sample further. Thus, the title of this paper might be inappropriate. Should it be modified?.

**Response:** Thank you for your thoughtful comments and suggestions. We have added the clarification of the representativeness of the resurvey sample (line 135-137, page 6-7) as “*Two periodic resurveys were conducted in 2008 and during August 2013 to September 2014, on approximately 5% of randomly chosen surviving participants using administrative unit as the basic sampling unit. The second re-survey was a representative sample of baseline sample.*”

In addition, we have revised the title to “Metabolically healthy obesity, transition to unhealthy metabolic status and vascular disease in Chinese adults: a prospective cohort study”.

2. Due to the small number of resurvey sample, the numbers of follow-up events of different vascular diseases in different categories of healthy or unhealthy overweight or obese participants were few. So, the grouping of overweight and obesity into one category when assessing the transition to healthy or unhealthy metabolic obesity was suggested.

**Response:** In the association analysis of the transition of metabolic health and obesity and cardiovascular diseases, we have already combined overweight and obese participants into one group. Detailed information is shown in Table 3 and Figure 4.

3. Please further strengthen that even healthy overweight or obesity state could also result in diabetes and other related metabolic disorders in discussion. So, even healthy overweight of obesity should also be paid attention to, to monitor glyceamia, blood pressure, and lipid profile, etc. And, healthy lifestyle intervention should be enhanced. Because the abnormalities of those indexes would indicate increasing CVDs risks.

**Response:** Thank you for your thoughtful suggestions. We have further strengthed the discussion accordingly. We have added related information in the discussion (line 423-430, page 19).

“*Our study suggests that recommendations for vascular risk prevention should highlight the importance of maintaining metabolic health regardless of body weight, in addition to the current focus on the treatment of metabolic disorders. Furthermore, previous studies showed that MHO participants also had higher risk of diabetes, our and previous studies further suggests that healthy overweight or obesity should also paid attention to maintaining metabolic health and monitoring glycemia, blood pressure, and lipid profile. Healthy lifestyle intervention should be enhanced among high risk population to prevent cardiometabolic disease*”

**Reviewer 1**

**Comments to the Author**

The authors have addressed my concerns and I now recommend publication.

**Response:** We thank the reviewer for the recommendation.

**Reviewer 2**

**Major comment:**

I cannot see where the authors have indeed addressed previous comments. The main limitations of this analysis remain as they had been:

1. The study findings are largely confirmatory - I am not convinced that providing regional data from China is creating a substantial novelty. Why would previous studies on MH and transitions not be largely generalizable to Chinese populations?

**Response:** We agree that several previous studies reported MHO individuals had higher risk of CVD. However, there is large variation the prevalence of obesity and metabolic disorders. MHO prevalence seemed to be higher in population from Asia than Caucasian or multi-ethnic origin[1]. Importantly, there is no large prospective study on transition of metabolic healthy status, and its association with incident cardiovascular disease, especially subtypes such as stroke, major coronary events, and ischemic heart disease in Asian people including Chinese. In addition, this is a large scale national wide representative cohort study, results from which may be generalizable to the whole population, and be of public health significance.

2. The sample with follow-up information on MH status and obesity is small. The combination of overweight and obese into one group doesn't allow to evaluate transition effects from MH in obesity.

**Response**: Thank you for your thoughtful comments and suggestions. We acknowledge that the sample with follow-up information on MH status and obesity is not large enough. We have added the limitation that “*due to low number of cases recorded in participants of the second re-survey, transition effects of obesity from metabolic healthy to unhealthy status couldn’t be evaluated with combination of overweight and obesity and CIs for HRs in the second re-survey and the transition were large. Further large prospective cohort study is required to confirm the observations in the present study*” (line 447-453, page 20).

3. Effect estimates are largely imprecise for analyses using the second survey as baseline for the most relevant group (MHO). The argument of the authors that these data are used to "validate" results from the baseline assessment isn't convincing - this is only possible for the least interetsing group (it is well established that individuals with obesity plius multiple cardiovascular risk factors are at higher risk compared to normal-weight health inmdividuals!)

**Response**: Thank you for your thoughtful comments. Please find our response to the abovementioned concern regarding sample size of resurvey. We clarify that the main objective of using the second survey was to examine the transition of healthy metabolic status and its association with cardiovascular disease. This is also the strength of our study. We have reworded the sentences accordingly (line 289-291, page 13-14). “*In the second re-survey, the results were similar. MHO individuals had higher risk of IHD, however, the CIs for MVE, MCE, and stroke were large. MUO individuals had higher risk of MVE, stroke and IHD.*”

In addition, we have revised the discussion on page 20 as “*It is worth noting that the results from the analysis in participants of the second re-survey were similar with those from baseline data, suggesting the robustness of our main findings*.”

4. I also don't agree with the authors notion, that it is not well established that MUH is related to higher risk across different BMI categories. While it is certainly easy to pick individual studies which might not have shown this, meta-analyses of cohort stdueis using the metabolic syndrome don't support this argument.

**Response**: Thank you for pointing out this key issue. We have corrected the words in the discussion accordingly (line 371-374, page 17).

“*Our findings show that the CVD risk of metabolically unhealthy individuals was much higher than that of metabolically healthy individuals across BMI categories, which were consistent with the results of meta-analysis[2] and did not support previous findings that metabolic status was not more valuable than BMI in identifying individuals at risk for CVD[3-5]*”

5. It is unfortunate that the autors did not attempt to evaluate alternative definitions of MH/MUH merely based on the argument that absence of any risk factor was extremly rare among obese. Many MH definitions used previously exclude WC (given the high correlation with BMI this measure isn't very informative to subgroup obese) for example.

**Response**: Thank you for your thoughtful comments and suggestions. We have added sensitivity analyses using an alternative MH definition excluding waist circumference criterion as suggested (line 256-259, page 12) in S1 Table and the results did not change.

**Reviewer 3**

**Comments to the Author**

Gao et. al have revised their manuscript examining the association of obesity (+/- metabolic healthy) and major cardiovascular events overall and by subtype in 500,000 Chinese adults across 10 regions (urban and rural). This is a very nicely done study and the authors have been very responsive to all suggestions--the abstract is clear and succinct and the introduction and discussion have been markedly improved. I have no additional major suggestions at this time and believe that this is a very timely much-needed addition.

**Response:** We thank the reviewer for the supportive comments.

**Major comment:**

In terms of the tables and figures, I find Table 1 a bit overwhelming and wonder if the baseline survey data could be presented here and the re-survey moved to the supplement and similarly for Table 2.

**Response**: Thank you for your thoughtful suggestions. We have revised it accordingly.

**Reference**

1.	Rey-Lopez JP, de Rezende LF, Pastor-Valero M, Tess BH. The prevalence of metabolically healthy obesity: a systematic review and critical evaluation of the definitions used. Obesity reviews : an official journal of the International Association for the Study of Obesity. 2014;15(10):781-90.

2.	Eckel N, Meidtner K, Kalle-Uhlmann T, Stefan N, Schulze MB. Metabolically healthy obesity and cardiovascular events: A systematic review and meta-analysis. European journal of preventive cardiology. 2016;23(9):956-66.

3.	Hinnouho GM, Czernichow S, Dugravot A, Nabi H, Brunner EJ, Kivimaki M, et al. Metabolically healthy obesity and the risk of cardiovascular disease and type 2 diabetes: the Whitehall II cohort study. European heart journal. 2015;36(9):551-9.

4.	Li L, Chen K, Wang AP, Gao JQ, Zhao K, Wang HB, et al. Cardiovascular disease outcomes in metabolically healthy obesity in communities of Beijing cohort study. International journal of clinical practice. 2018:e13279.

5.	Thomsen M, Nordestgaard BG. Myocardial infarction and ischemic heart disease in overweight and obesity with and without metabolic syndrome. JAMA internal medicine. 2014;174(1):15-22.
