# Supplementary material for: Metabolically healthy obesity, transition to unhealthy metabolic status, and vascular disease in Chinese adults: A cohort study
Source: PLoS Med. 2020 Oct 30;17(10):e1003351. doi: 10.1371/journal.pmed.1003351 (PMC7598496; doi:10.1371/journal.pmed.1003351)
Supplement: Attachment — Submitted filename: Response letter.docx [file pmed.1003351.s015.docx]

Dear Editor,

Manuscript PMEDICINE-D-20-00639R3

**Metabolically healthy obesity, transition to unhealthy metabolic status and vascular disease in Chinese adults: a cohort study**

Thank you for your comments on the above manuscript and for the opportunity to revise it. We have taken the editor’s comments and suggestions into careful consideration and revised the manuscript accordingly. On the following pages, please find our point-to-point responses to the editor’s concerns in the order that they were originally listed, and details of the pages on which the changes have been made.

Sincerely,

Prof. Tao Huang,

Department of Epidemiology & Biostatistics,

School of Public Health, Peking University, China.

38 Xueyuan Road, Bejing, 100191 China

Phone: (86) 010-82801528

Email: huangtaotao@pku.edu.cn

**Response to Editor’s comments**

We have revised the manuscript in light of helpful comments from editors. Our responses to the specific comments raised are provided below, along with the line numbers of the related text in the revised manuscript.

**Editor**

**Major comment:**

1. The author summary needs to be in bulleted form and with 3 headings. Please do look at other published Research articles for guidance. It should also be written for the non-specialist and not repeat the abstract.

**Response:** Thank you for your suggestions. We have revised the author summary accordingly.

2. In the main text, please include a space between the last letter of the word and the following square bracket for refs – For example, instead of 17 million deaths annually[1], it should read 17 million deaths annually [1]. Correct throughout, please.

**Response:** We have revised it accordingly.

3. Please ensure all questionnaires are provided as Supplementary Files and translated where necessary.

**Response:** We have added baseline and the 2^nd^ re-survey questionnaires in Supplementary Files (S2 Text).

4. At line 334, Please mention their Chinese population and avoid "a little lower risk" as this is too vague. Say what the risk is.

**Response:** Thank you for your thoughtful comments and suggestions. We have revised it in the discussion (line 343-345) as *“Our findings showed that in Chinese adults, MHO individuals had an 8% higher risk of developing MVE, 34% higher risk of IHD, and 11% higher risk of stroke, with no association found with MCE.”*

5. Please remove the word "prospective" from the title (I believe that this is a retrospective analysis of a prospectively gathered dataset).

**Response:** We have removed the word "prospective" from the title.

**Production issues:**

**FIGURES and GRAPHICS:**

1. Please note that your figures do not currently comply with our publication requirements. For further instructions to assist with your Figure revisions, please refer to our Figure Guidelines at: http://journals.plos.org/plosmedicine/s/figures.

If you need assistance, please contact us directly at graphics@jjeditorial.com so we can further help with this matter. Please send any relevant source files and tell us what application(s) and what operating system you are using.

- Please remove the figure numbers from Figures 1, 2, 3, and 4. These should only be present in the body of your manuscript and will appear alongside your main figure when published.

- Some text in Figure 1 appears pixelated/low quality. Please remake this part of the figure using the original source, using the PLOS figure guidelines to ensure quality is preserved [http://journals.plos.org/plosmedicine/s/figures].

- Please crop Figures 1, 2, and 3 to remove the whitespace around the figures themselves. PLOS recommends a 2-point whitespace border, but more will cause the figures to be incorrectly placed in the published PDF.

**Response:** We have removed the figure numbers from Figures 1, 2, 3, and 4. We have revised the text in Figure 1 and cropped Figures 1, 2, and 3 accordingly.

2. Please note that Figures 1-4 are currently split into multiple parts in separate files. Please either combine the parts into four separate figures with lettered panels, or renumber your figures as whole parts (i.e. Fig 1A becomes Fig 1, Fig 1B becomes Fig 2 etc.), ensuring that all relevant legends and in-text citations have been updated accordingly.

**Response:** We have combined the parts into one page with lettered panels accordingly.

**METADATA:**

1. Please ensure your short title is correct in the submission form, including symbols, italicisation etc. Your short title will be typeset from the submission form directly.

**Response:** We have checked the short title is correct.

2. Please provide a detailed Financial Disclosure statement in the submission form. Include grant numbers and URLs as appropriate, as well as the initials of the authors who received the funding. This is published with the article, therefore it should be completed in full sentences and contain the exact wording you wish to be published.

Please add the statement 'The funders had no role in study design, data collection and analysis, decision to publish, or preparation of the manuscript' to the end of your Financial Disclosure statement on the submission form. If this statement is inaccurate, please describe the role the funders had.

If you received no funding, please add the statement 'The author(s) received no specific funding for this work' to your submission form.

**Response:** We have added the Financial Disclosure statement in the submission form.

3. In accordance with PLOS' data policy, please ensure that your Data Availability Statement in the submission form clearly identifies how readers can access your data. Note that it will be typeset as it is written so please ensure it is in complete sentences and appears as you would like it to in the published version.

For details on our data policy, please see http://journals.plos.org/plosmedicine/s/data-availability

**Response:** We have checked Data Availability Statement in the submission form. *“Details of how to access China Kadoorie Biobank data and details of the data release schedule are available from www.ckbiobank.org/site/Data+Access”*

**ARTICLE FILE:**

1. Please indicate the corresponding author on the title page with an asterisk (*) and include the corresponding author's email address. Only an asterisk and the email address itself are required.

**Response:** We have revised the information of corresponding author accordingly.

2. Please remove author degree titles from the byline of your manuscript file.

**Response:** We have removed author degree titles from the byline accordingly.

3. Please ensure that all authors have at least one affiliation listed on the title page and that all listed affiliations are attributed to at least one author. Affiliations should be listed in ascending numerical order in the affiliation list and should match what is provided in the Authors Information in the submission form.

Affiliations should include an institution, a city, and a country (no postcode). US affiliations need the state as well, written out in full (with no abbreviations).

**Response:** We have checked the affiliation of authors and made revisions accordingly.

4. Please ensure that all author names are spelled correctly in the manuscript file and the submission form, including the use of a middle name/initials, and any special characters (accents, umlauts, etc.). Please place first/given names before last/family names. We ask that you ensure they match verbatim. Please be aware that correct spelling and formatting of author names in the article file is vital for accurate indexing in PubMed.

**Response:** We have checked all author names.

5. As your paper involves contributions from China Kadoorie Biobank (CKB) Collaborative Group, please format your paper as follows:

1) Please list China Kadoorie Biobank (CKB) Collaborative Group as an author in the byline.

2) If you wish to include the names of individual group members in the manuscript, you may list them in the Acknowledgments section.

Please be aware that group author members are no longer indexed in PubMed as collaborators.

**Response:** We have listed CKB Collaborative Group as an author in the byline and listed them in the Acknowledgments section.

6. Please ensure that your Ethics Statement is available in the Methods section of your manuscript in its entirety.

**Response:** We have checked Ethics Statement in the Methods section (line 154-156).

7. We have noted that you have the following present in your manuscript: Financial Disclosure/ Competing Interests statement/ Author Contributions/ Data Availability Statement

We have removed these section from your manuscript file as this information is automatically pulled from the submission form. Please make sure that the information in the submission form is correct.

**Response:** We have removed these sections from manuscript and added the information in the submission form.

**REFERENCES:**

1. Please make sure all references are cited in ascending numerical order in the text.

**Response:** We have checked the references.

**SUPPORTING INFORMATION:**

1. Please ensure that all Supporting Information files are clearly labeled in the file inventory. i.e. S1_Fig.tif, S1_Table.xlsx, S1_Text.doc, S1_Data.xlsx, S1_PRISMA_Checklist.docx, etc.

Each file type should be numbered starting at 1. Please also make sure that the file description matches the file name (e.g. S1_Fig.tif is listed as S1 Fig).

**Response:** We have checked the supporting information file.

2. Please ensure that each supporting information file has a legend at the end of the manuscript file, after the Reference list.

**Response:** We have checked the supporting information file and made revisions.

3. Please note that your submission cannot contain any references to generic "supporting information" files and instead must refer to specific files using our naming convention (S1 Fig, S1 Table, S1 Text, S1 Data, etc.)

**Response:** We have checked the supporting information files.

**Editor2:**

1. Please indicate the corresponding author on the title page with an asterisk (*) and include the corresponding author's email address. Only an asterisk and the email address itself are required.

**Response:** We have indicated the corresponding author on the title page with an asterisk (*) and include the corresponding author's email address.

2. Please ensure that all main figures are referenced in the manuscript as Fig 1, Fig 2, etc. (including capitalization), rather than Figure 1, Figure 2, etc. Please note, however, that the file names themselves must not include the space, i.e. Fig1.tif, Fig2.tif. Please ensure that all main figure files are clearly labeled as such in the File Inventory.

**Response:** We have checked that all main figures are referenced in the manuscript as Fig 1, Fig 2, etc. We have revised the file names without the space.

3. Please ensure that all Supporting Information files are clearly labeled in the file inventory. i.e. S1_Fig.tif, S1_Table.xlsx, S1_Text.doc, S1_Data.xlsx, S1_PRISMA_Checklist.docx, etc.

Each file type should be numbered starting at 1. Please also make sure that the file description matches the file name (e.g. S1_Fig.tif is listed as S1 Fig).

**Response:** We have checked that all Supporting Information files are clearly labeled in the file inventory and each file type is numbered starting at 1. We have checked that the file description matches the file name.

4. We have noted that you have the following present in your manuscript: Financial Disclosure (Funding)

We have removed these section from your manuscript file as this information is automatically pulled from the submission form. Please make sure that the information in the submission form is correct.

**Response:** We have checked that the information in the submission form is correct.

5. Figure 1 looks like it was originally at a lower resolution and will appear blurry when published. This error cannot be fixed by simply increasing the resolution of your figures with image editing software. Instead, please carefully review the PLOS Figure Guidelines and provide figure files that were originally created at a minimum of 300 pixels/inch (ppi) and that are sized between 6.68cm - 19.05cm wide, and no more than 22.225cm tall (excluding any white space around your artwork). Additionally, please ensure your figures are in RGB colour mode.

**Response:** We have revised Figure 1 with more pixels accordingly.

6. Please resave your Figure 1 using LZW compression (loss-less) in order to reduce the file size by over 75% and re-upload.

In Photoshop: File→Save As. Save as TIFF, Image Compression set to LZW, Pixel Order set to Interleaved, Byte Order set to IBM PC.

In GIMP: File→Save As. Click the + sign next to Select File Type (By Extension). From the menu that appears, select TIFF. Click Save. Set Compression to LZW. If you're prompted about layers in the file, select Flatten Image.

**Response:** Thank you for your instruction and we have revised Figure 1 accordingly .

7. Figures 2-4 have been modified to meet with formatting requirements. Please review the revised figures to ensure that they meet with your approval. Please note that if you approve of these revised figures, you do not need to upload these to the system.

**Response:** We have approved of these revised figures.
